# Photo/electro-mediated radical cascade functionalization/cyclization reactions involving *N*-acryloyl 2-aryl indoles/benzimidazoles

**DOI:** 10.1039/d5ra03878b

**Published:** 2025-09-05

**Authors:** Fatemeh Doraghi, Amirali Abbasi, Nooshin Zomorodiyan, Shahab Kermaninia, Reza Hassanzadeh Dostkoh, Bagher Larijani, Mohammad Mahdavi

**Affiliations:** a Endocrinology and Metabolism Research Center, Endocrinology and Metabolism Clinical Sciences Institute, Tehran University of Medical Sciences Tehran Iran momahdavi@tums.ac.ir; b School of Chemistry, College of Science, University of Tehran Tehran Iran; c Faculty of Science, Islamic Azad University Karaj Branch Karaj Iran; d Pharmaceutical and Heterocyclic Chemistry Research Laboratory, Department of Chemistry, Iran University of Science and Technology Tehran Iran

## Abstract

Indolo/benzimidazo-isoquinoline scaffolds are frequently found in many natural products, pharmaceuticals, and organic materials. Owing to their prominent properties, in recent years, numerous studies have been performed on the synthesis of indolo/benzimidazo-isoquinoline derivatives *via* photo-, and electro-promoted functionalization/cyclization reactions of *N*-acryloyl 2-aryl indoles/benzimidazoles. In this review, we describe these fascinating transformations and discuss their mechanistic insights.

## Abstract 2

This review highlights the synthesis of pharmaceutically important indolo/benzimidazo-isoquinoline derivatives *via* photo-, and electro-mediated functionalization/cyclization reactions of *N*-methacryloyl 2-aryl indoles/benzimidazoles.

## Graphical abstract

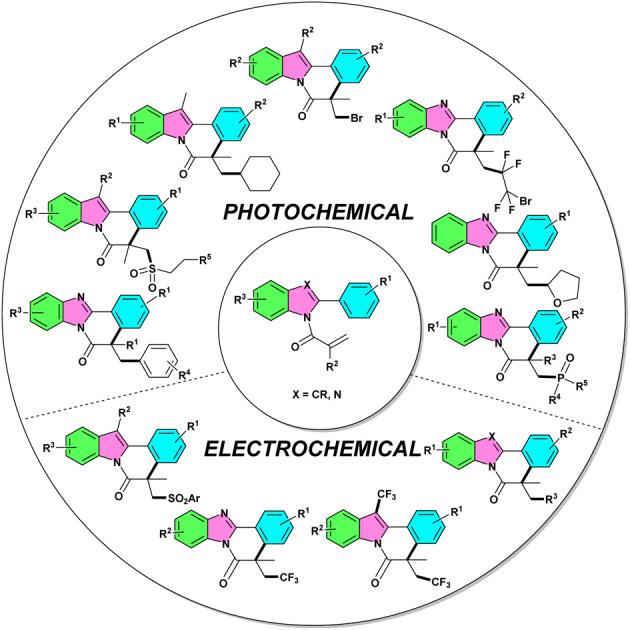

## 1 Introduction

**Fig. 1:**
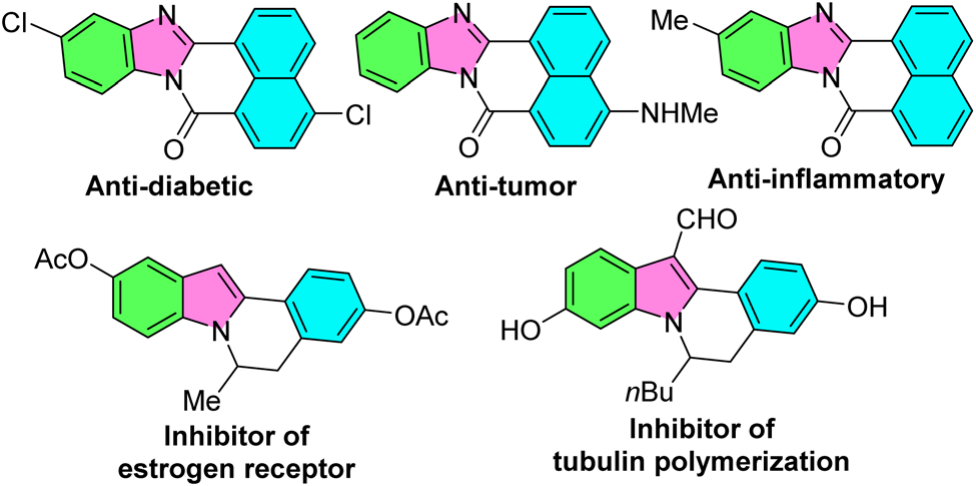
Representative natural products and biologically active molecules involving indolo/benzimidazo-isoquinoline cores.

Nitrogen-containing heterocycles constitute an important and promising class of organic chemicals and are an integral part of medicinal chemistry.^1–3^ In particular, indolo[2,1-*a*]isoquinolin-6(5*H*)-ones and benzimidazo-[2,1,*a*]isoquinoline-6(5*H*)-ones have attracted a great deal of attention due to their fascinating bioactivities, such as inhibitor of estrogen receptor, inhibitor of tubulin polymerization,^4^ cytostatic agents,^5^ anti-tumor,^6^ anti-inflammatory, antimicrobial,^7^ antifungal,^8^ anti-HIV-1,^9^ cardiovascular agents,^10^*etc*. Some of these biologically active molecules including indolo/benzimidazo-isoquinoline cores are shown in Fig. 1.

Classic procedures for the preparation of indolo/benzimidazo fused isoquinolinones suffer from difficulty in accessing raw materials, long and tedious reaction steps, harsh conditions and poor tolerance of functional groups.^11,12^ Consequently, developing atom- and step-economical synthetic methodologies for synthesizing indolo[2,1-*a*]isoquinolin-6(5*H*)-ones and benzimidazo[2,1,*a*]isoquinoline-6(5*H*)-ones has received much attention in both academia and industry.

Over the past few years, various methodologies, including metal-catalyzed reactions^13–19^ and metal-free reactions,^20–25^ have been developed for the synthesis of indolo/benzimidazo [2,1-*a*]isoquinolin-6(5*H*)-ones starting from *N*-acryloyl 2-aryl indoles/benzimidazoles. Recently, photo- and electro-mediated syntheses have emerged as powerful, and environmentally friendly strategies for a wide range of organic transformations.^26–28^ Especially, the synthesis of structurally diverse polycyclic indole/benzimidazole fused isoquinolines under photochemical and electrochemical conditions has been well developed in the last few years. In this regard, many research groups introduced practical and direct approaches based on radical cascade functionalization/cyclization reactions of *N*-methacryloyl 2-aryl indoles/benzimidazoles in the presence of transition metal catalysts, organocatalysts or in the absence of catalyst. Almost all reactions proceed *via* photo- or electro-triggered radical formation, followed by a radical addition/cyclization/aromatization sequence. In fact, such transformations provide green, and mild conditions, as well as ease of operation.

Considering the synthetic utility and prevalent biological activities of indolo/benzimidazo fused isoquinolinones, in this review article, we cover various photo/electro-mediated radical functionalization/cyclization reactions of *N*-methacryloyl 2-aryl indoles/benzimidazoles since 2019. The scope of the reactions and the challenging mechanisms are also described.

## 2 Radical cascade functionalization/cyclization of *N*-methacryloyl 2-aryl indoles/benzimidazoles

### 2.1 Photo-mediated metal-catalyzed reactions of *N*-methacryloyl 2-aryl indoles/benzimidazoles

As a consequence of their importance, the conventional condensation strategies are efficient for the construction of indolo[2,1-*a*]isoquinolin-6(5*H*)-ones and benzimidazo[2,1,*a*]isoquinoline-6(5*H*)-ones.^11^ However, these reactions suffer from tedious pre-functionalization steps, harsh reaction conditions as well as practical inconvenience. Gratifyingly, transition metal catalysis systems have been realized as promising alternatives for the synthesis of such important polycycles.^19,29,30^

In radical cascade functionalization/cyclization reactions, metal complexes have an inherent ability to form stable, long-lived excited states, which makes them more suitable for specific catalytic applications, especially in photoredox catalysis.

#### 2.1.1 Ir-catalyzed reactions of *N*-methacryloyl 2-aryl indoles/benzimidazoles

##### 2.1.1.1 Acylation/cyclization

**Scheme 1:**
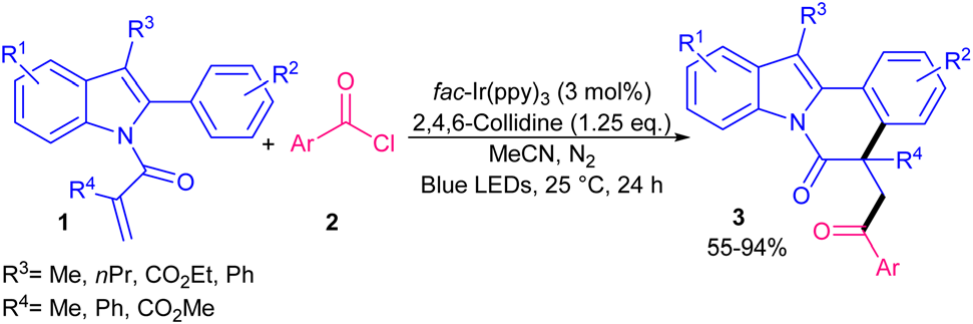
Ir-catalyzed acylation/cyclization of *N*-methacryloyl 2-phenyl indoles with acyl chlorides.

**Scheme 2:**
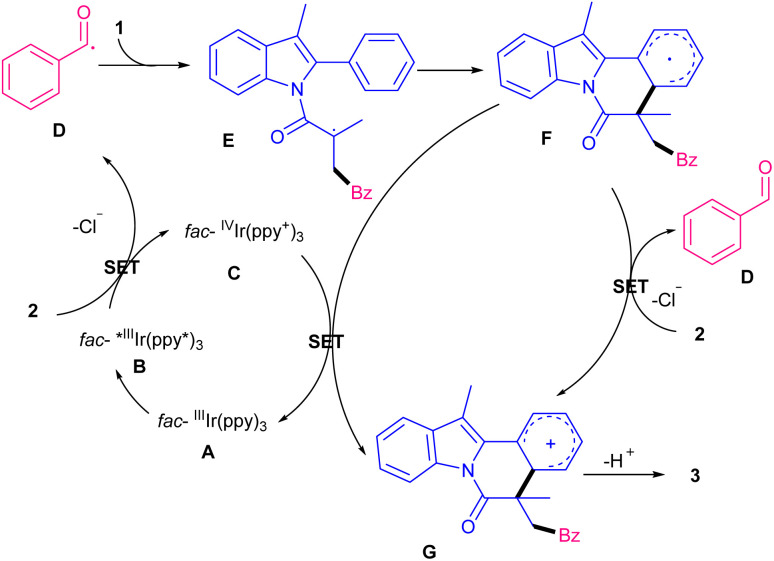
Catalytic cycles for Ir-catalyzed acylation/cyclization of *N*-methacryloyl 2-phenyl indoles with acyl chlorides.

**Scheme 3:**
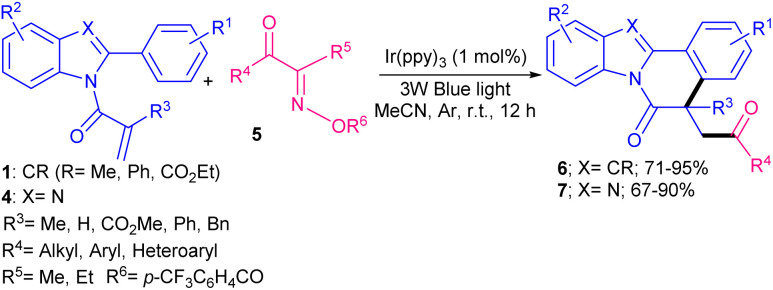
Ir-catalyzed acylation/cyclization of *N*-methacryloyl 2-phenyl benzimidazoles/indoles with acyl oximes.

In 2019, Xu and co-workers developed a new strategy for radical cascade acylation/cyclization of *N*-methacryloyl 2-aryl indoles 1 as radical acceptors with acyl chlorides 2 as acyl radical precursors (Scheme 1).^31^ For this purpose, *fac*-Ir(ppy)_3_ was selected as an optimal photocatalyst in the presence of blue LEDs to obtain indolo[2,1-*a*]isoquinolines in good to high yields. Various aroyl/heteroaroyl chlorides bearing electron-donating and electron-withdrawing groups at the aryl rings were compatible, while alkyl acid chloride and cinnamoyl chloride were not feasible. As shown in the mechanism, *fac*-Ir(iii)(ppy)_3_A was converted to *fac*-Ir(iii)(ppy)_3_* B. The following SET reaction with benzoyl chloride 2 generated the acyl radical D after the release of chloride. In the meantime, the oxidized *fac*-Ir(iv)(ppy)_3_C was generated. Then, D added to 1 to produce the α-acyl radical E, which underwent cyclization reaction to form radical F. Subsequently, the oxidation of F with *fac*-Ir(ppy)_3_C provided cation G and *fac*-Ir(ppy)_3_A. Alternatively, F was oxidized by benzoyl chloride 2 to render cation G, along with the regeneration of acyl radical D. Finally, G was deprotonated to furnish product 3 (Scheme 2). Another acylation/cyclization reaction of *N*-methacryloyl 2-phenyl benzimidazoles/indoles was reported in the presence of the same Ir(ppy)_3_ catalyst (Scheme 3).^32^ Other photocatalysts, such as eosin Y-Na_2_, eosin Y, and Ru(bpy)_2_Cl_2_ also afforded the target product, albeit in lower yields (21–51%). Substituted acyl oximes 5 were used as acylating reagents to generate acyl radicals for the next radical addition/cyclization to access indolo- and benzimidazo-[2,1,*a*]isoquinoline-6(5*H*)-one products 6, 7. *N*-Acryloyl benzimidazoles containing electron-donating groups or electron-withdrawing groups at the *para*-, and *ortho*-position of the 2-phenyl moiety displayed good selectivity and reactivity compared to the *meta*-substituted group. The reaction also did not occur when the phenyl was replaced with benzyl.

**Scheme 4:**
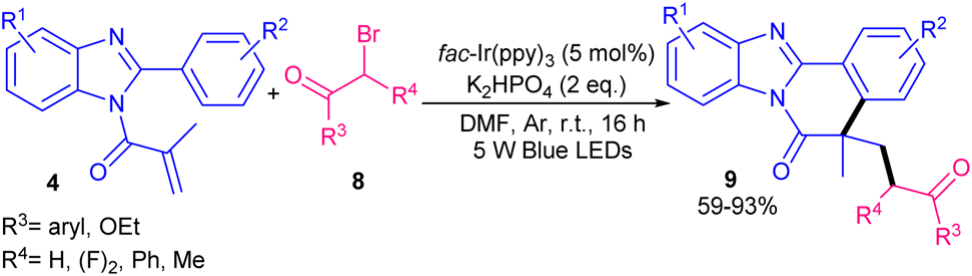
Photocatalysis reaction of *N*-methacryloyl 2-phenyl benzimidazoles with α-carbonyl alkyl bromides.

*fac*-Ir(ppy)_3_ was also used by Gao and his team in the reaction of *N*-methacryloyl 2-phenyl benzimidazoles 4 with α-carbonyl alkyl bromides 8 (Scheme 4).^33^ Other common photocatalysts like Ir[dF(CF_3_)ppy]_2_(dtbpy)PF_6_, Ru(ppy)_3_Cl_2_ and eosin Y showed inferior catalytic activities. α-Carbonyl alkyl bromides with electron-donating and electron-withdrawing groups all showed good reactivities at the *para*-, *meta*-, and *ortho*-positions (62–93% yield). Although strong electron-attracting ability of NO_2_ group at the *para*-position of the benzene ring destabilized the α-carbonyl alkyl radical, thus afforded the product in 32% yield. Some of the obtained benzimidazo[2,1-*a*]isoquinolin-6(5*H*)-ones revealed good potential against two cancer cell lines. A similar mechanism to sulfonylation/cyclization reaction was suggested for this transformation, involving the formation of alkyl radical from the interaction of the iridium catalyst, α-carbonyl alkyl bromide and visible light, followed by trapping by *N*-methacryloyl 2-phenyl benzimidazole. Sequential radical addition cyclization, oxidation and deprotonation afforded the final products.

##### 2.1.1.2 Alkylation/cyclization

**Scheme 5:**
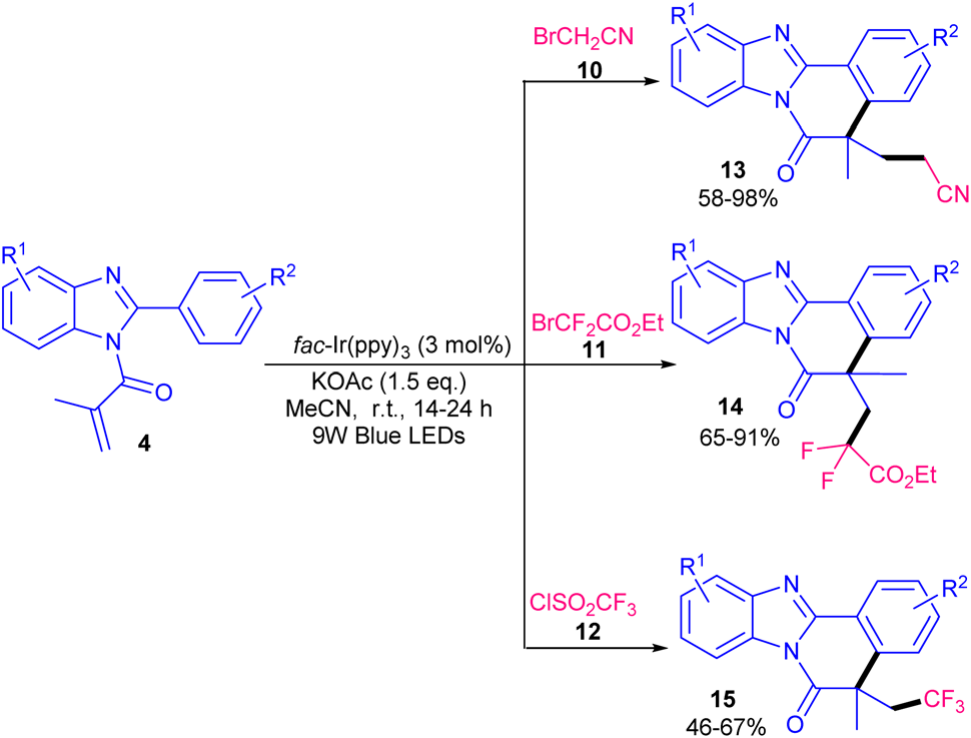
Ir-catalyzed photo-induced reaction of *N*-methacryloyl 2-phenyl benzimidazoles with radical precursors.

**Scheme 6:**
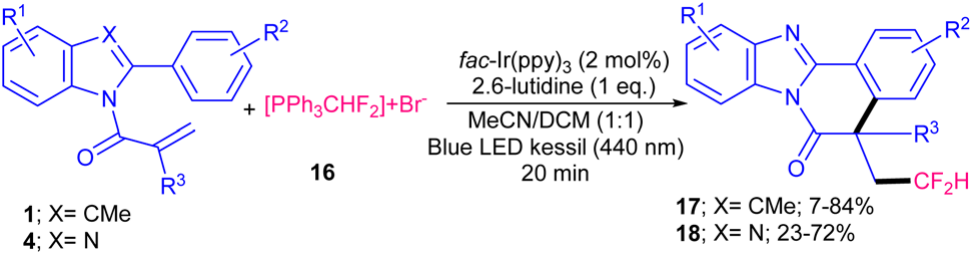
Ir-catalyzed difluoromethylation/cyclization of *N*-methacryloyl 2-phenyl benzimidazoles/indoles.

**Scheme 7:**
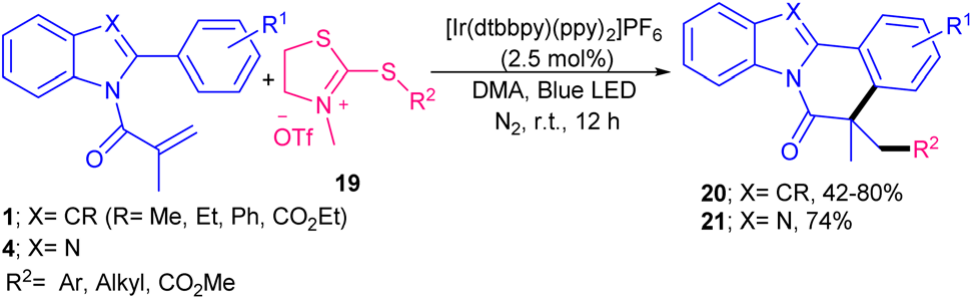
Ir-catalyzed alkylation/cyclization of *N*-methacryloyl 2-phenyl indoles/benzimidazole.

In 2020, Guan, He and co-workers developed a photocatalytic system based on an iridium catalyst for the radical cascade cyclization of *N*-methacryloyl 2-phenyl benzimidazoles 4 with different radical precursors 10–12 (Scheme 5).^34^ Several free radical initiators, such as bromoacetonitrile 10, ethyl difluorobromoacetate 11, and trifluoromethane sulfonyl chloride 12 reacted well under these photocatalytic conditions, leading to the corresponding products 13–15 in moderate to excellent yields. Other radical precursors like diethyl bromomalonate gave 63% yield of product, while perfluoroiodide and diethyl bromide difluoromethane diphosphonate were not workable. TEMPO and BHT as radical scavengers and HRMS analysis confirmed the involvement of a radical pathway. Since the reaction proceeded slightly under dark conditions, the authors suggested a free radical chain propagation process. In addition, the emission spectrum of the lamp and the UV-Visible absorption spectra of substrates revealed that *fac*-Ir(ppy)_3_ is the only light-absorbing species in the reaction. The mechanism involved photo-triggered Ir-catalyzed initial alkyl radical formation, radical addition, intramolecular cyclization, and final aromatization. An iridium catalyst was used for difluoromethylation/cyclization of *N*-methacryloyl-2-phenyl benzimidazoles/indoles (Scheme 6).^35^ In this regard, Arseniyadis and co-workers employed 2 mol% of *fac*-Ir(ppy)_3_ as the catalyst, and 1 equiv. of 2,6-lutidine as the base in the reaction of *N*-methacryloyl 2-phenyl benzimidazoles 4 or *N*-methacryloyl 2-phenyl indoles 1 with PPh_3_CHF_2_^+^Br^−^16. Screening of photocatalysts, such as 4CzlPN, eosin Y and Ru(bpy)_3_ did not yield the desired product. Other bases, such as DIPEA, NE*t*_3_, K_2_CO_3_ and DABCO also provided the product albeit in lower yields (35–67%). Electron-rich benzimidazoles and indoles displayed higher reactivities than electron-poor ones, especially strong electron-withdrawing NO_2_ group. A series of CHF_2_-containing benzimidazo- and indolo[2,1-*a*]isoquinolin-6(5*H*)-ones were well synthesized in modearte to good yields. Very recently, Xu and co-workers employed 2-mercaptothiazolinium salts 19 as an alkyl radical source for alkylation/cyclization of *N*-acryloyl 2-aryl indoles 1 (Scheme 7).^36^ Among, various solvents; MeCN, THF, DCM, NMP, DMF, DMSO and DMA, the polar solvents, especially, DMA showed better efficiency. Screening of various photocatalysts; CzIPN, *fac*-Ir(ppy)_3_, [Ru(bpy)_3_](PF_6_)_2_ and [Ru(bpy)_3_](Cl)_2_ indicated that none of them were as effective as [Ir(dtbbpy)(ppy)_2_]PF_6_. Thus, the reaction was performed in the presence of 2.5 mol% of [Ir(dtbbpy)(ppy)_2_]PF_6_ in DMA under visible light irradiation, no need for base or oxidant. Both the iridium catalysts and light were necessary for this alkylation/cyclization reaction to proceed. Various *N*-acryloyl 2-aryl indoles reacted well with 2-mercaptothiazolinium salts bearing alkyl, aryl or ester moieties affording alkylated indolo[2,1-*α*]isoquinolines 20 in moderate to high yields (42–80%). Besides, *N*-acryloyl 2-phenyl benzimidazole 4 was also showed good compatibility in this reaction system, yielding the corresponding alkylated benzimidazo[2,1-*α*]isoquinoline 21 in 74% yield.

##### 2.1.1.3 Carboxylation/cyclization

**Scheme 8:**
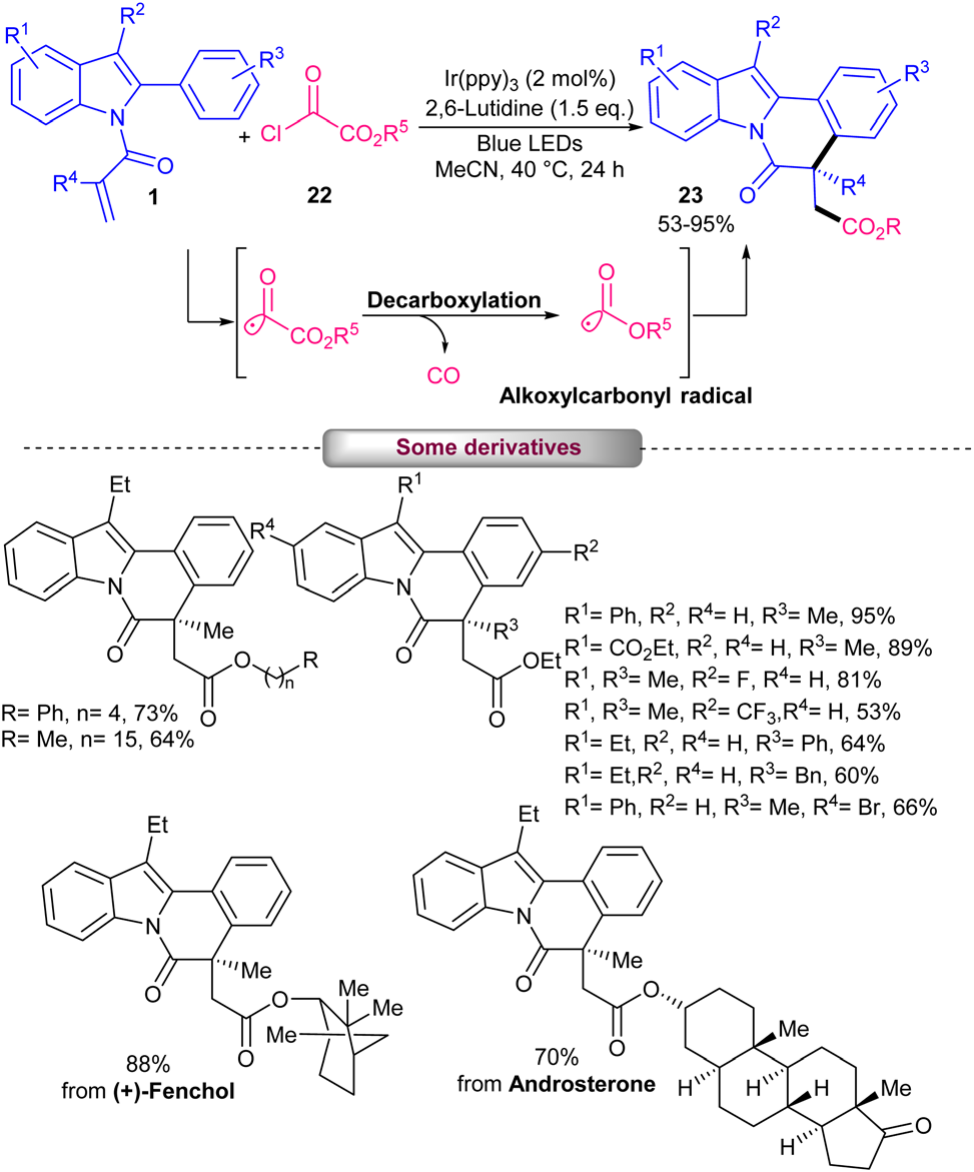
Ir-catalyzed alkoxycarbonylation/cyclization of 2-aryl *N*-acryloyl indoles with alkyloxalyl chlorides.

Iridium-catalyzed alkoxycarbonylation/cyclization of *N*-acryloyl 2-aryl indoles 1 with alkyloxalyl chlorides 22 was reported by Zhang and coauthors in 2022 (Scheme 8).^37^ Synthetic application of this method was showed by the gram-scale preparation of the product (0.57 gr, 79%). Also, the reduction of the carbonyl group, followed by the furan ring formation in indolo[2,1-*a*]isoquinoline was performed in the presence of LiAlH_4_ as a reductant agent. The authors also showed the role of heat in increasing product yield by performing the reaction in different temperatures (0 °C, 20 °C, and 40 °C). TEMPO as a radical scavenger proved the presence of the alkoxycarbonyl radical in the reaction media. A diverse range of *N*-acryloyl 2-aryl indoles containing electron-donating and electron-withdrawing substituents at the aryl rings could participate in the reaction with alcohol-derived alkyloxalyl chlorides. Additionally, the late-stage synthesis of some biologically active molecules containing indolo[2,1-*a*]isoquinolines was also possible in this method.

##### 2.1.1.4 Sulfonylation/cyclization

**Scheme 9:**
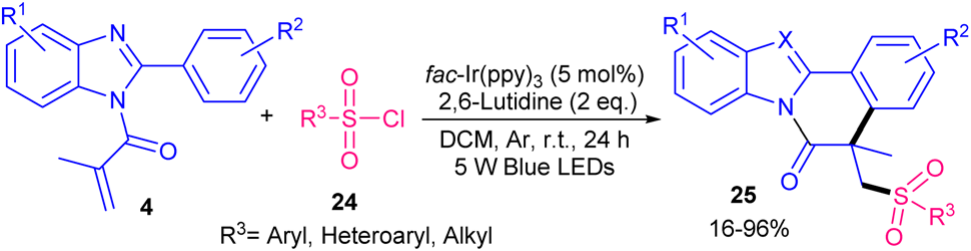
Ir-catalyzed reaction of *N*-methacryloyl 2-phenyl benzimidazoles with sulfonyl chlorides.

A step-economical tool for the sulfonylation/cyclization of *N*-methacryloyl 2-phenyl benzimidazoles 4 with sulfonyl chlorides 24 as a sulfonylating reagent was suggested by Gao ad co-workers (Scheme 9).^38^ For this purpose, *fac*-Ir(ppy)_3_ was used to generate sulfonyl radicals with the aid of visible light irradiation. Other photocatalysts, such as [Ir{dF(CF_3_ppy)}_2_(dtbbpy)] PF_6_, Ir(ppy)_2_(dtbbpy)(PF_6_), Ru(bpy)_3_Cl_2_ or eosin Y were not effective, giving trace amount of the product. These sulfonyl radical were trapped by *N*-methacryloyl 2-phenyl benzimidazoles. Subsequently, intramolecular cyclization, single electron oxidation and deprotonation produced sulfonylated indolo/benzimidazo[2,1-*a*]isoquinolin-6(5*H*)-ones. Both aryl sulfonyl chlorides and alkyl sulfonyl chlorides were found to be suitable in this radical cyclization reaction. The gram-scale synthesis of the product showed 80% yield. The reaction influenced by the electronic effects; the electron-donating substituents (R = *t*Bu, Me, OMe) at the *para*- and *meta*-position of the phenyl ring of *N*-methacryloyl 2-phenyl benzimidazoles resulted in higher yields than halogen groups. At last, the antitumor activity of the products were tested that showed good potential activities.

**Scheme 10:**
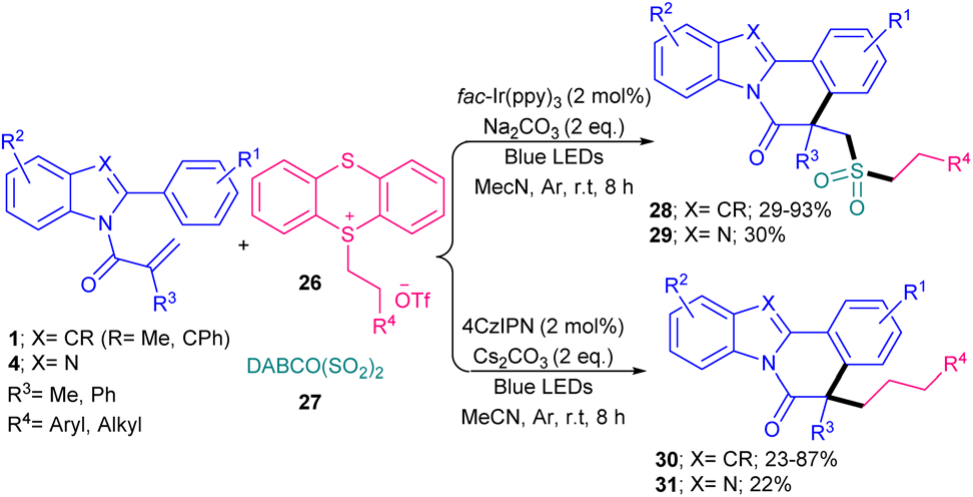
Ir-catalyzed sulfonylation/cyclization of *N*-methacryloyl 2-phenyl benzimidazoles/indoles with DABCO (SO_2_)_2_ and thianthrenium salts.

**Scheme 11:**
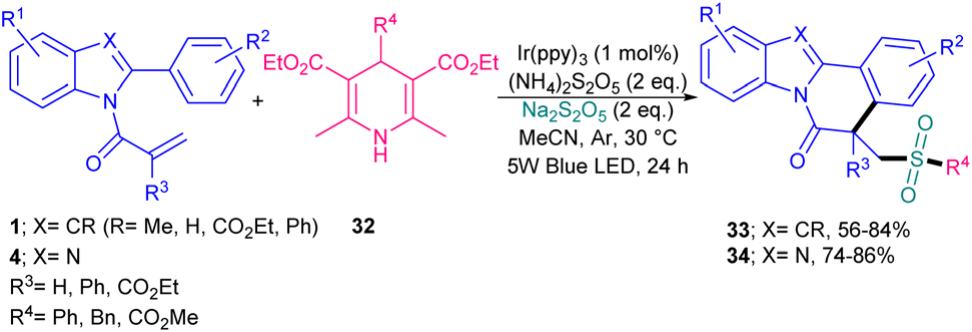
Ir-catalyzed sulfonylation/cyclization of *N*-acryloyl 2-aryl benzimidazole/indole with Hantzsch esters and Na_2_S_2_O_5_.

Sulfonylated indolo/benzimidazo [2,1-*a*] isoquinoline derivatives can be synthesized from the sulfonylation/cyclization of *N*-acryloyl 2-aryl indoles/benzimidazoles 1, 4 with DABCO (SO_2_)_2_27 and thianthrenium salts 26 (Scheme 10).^39^ A diverse range of functionalized indolo[2,1-*a*] isoquinolines bearing a alkyl/aryl/heteroaryl sulfonyl portion 28 were obtained in low to high yields (29–93%) using *fac*-Ir(ppy)_3_ as a photocatalyst and Na_2_CO_3_ as a base. The study of one derivative of benzimidazo[2,1-*a*] isoquinoline 29 showed 30% yield of product. Interestingly, this sulfonylation/cyclization reaction was also possible by changing the photocatalyst and base to 4CzlPN and Cs_2_CO_3_, respectively, leading to the corresponding indolo[2,1-*a*] isoquinolines 30 in 23–87% yields. While eosin Y and g-C_3_N_4_ did not yield the product. The inhibition of the both metal-catalyzed reaction and organocatalyst-catalyzed reaction in radical trapping experiments using TEMPO suggested radical routes for these transformations, and Stern–Volmer quenching experiments showed that SET starts the charge transfer of the excitation process. Many electron-withdrawing and electron-donating groups on the both substrates displayed good compatibility in the sulfonylation/cyclization reactions. Another three-component reaction between *N*-acryloyl 2-aryl indoles/benzimidazoles 1 and 4, Hantzsch esters 32, and Na_2_S_2_O_5_ (Scheme 11).^40^ In this method, Hantzsch esters as an alkyl precursor and Na_2_S_2_O_5_ as a SO_2_ surrogate incorporated in radical cascade cyclization, followed by SO_2_ insertion of *N*-acryloyl 2-aryl indoles/benzimidazoles. The radical mechanism was confirmed by using radical inhibitors; TEMPO, BHT, or 1,1-diphenylethen. Notably, only primary and secondary alkyl radicals could be incorporated in this reaction and a tertiary butyl radical did not result in the desired product. The use of the iridium photocatalyst, (NH_4_)_2_S_2_O_8_ and visible light was found to be necessary for this reaction to proceed. Organocatalyts, such as Na_2_-eosin Y, and eosin Y showed lower activities (14% and 59%) and Ru(bpy)_3_Cl_2_ led to 65% yield. In addition to LEDs, 36 W compact fluorescent light was also applicable for this cyclization, affording the product in 59%.

#### 2.1.2 Ru-catalyzed reactions of *N*-methacryloyl 2-aryl indoles/benzimidazoles

##### 2.1.2.1 Alkylation/cyclization

**Scheme 12:**
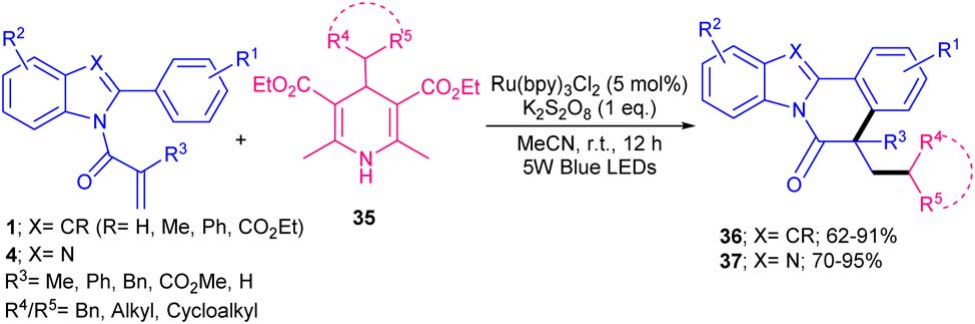
Ru-catalyzed alkylation/cyclization of *N*-methacryloyl 2-phenyl benzimidazoles/indoles.

**Scheme 13:**
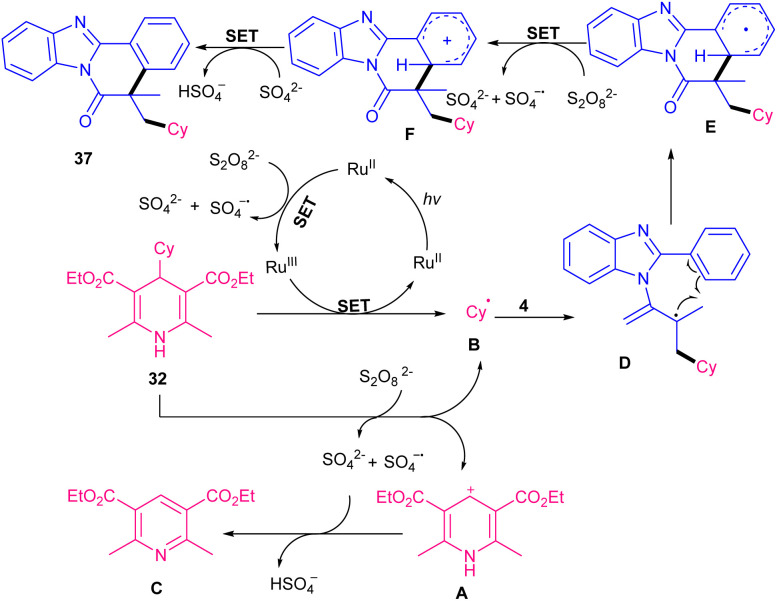
Rational mechanism for Ru-catalyzed alkylation/cyclization of *N*-methacryloyl 2-phenyl benzimidazole.

**Scheme 14:**
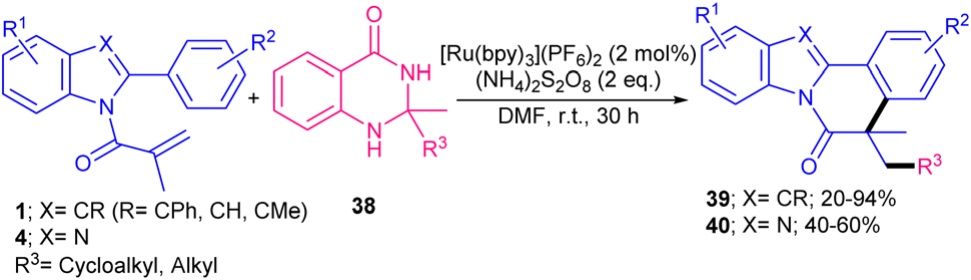
Ru-catalyzed alkylation/cyclization of *N*-methacryloyl 2-phenyl benzimidazoles/indoles.

The alkylation/cyclization of a wide range of *N*-acryloyl 2-aryl indoles and 1*N*-acryloyl 2-aryl benzimidazoles 4 were performed in the presence of Ru(bpy)_3_Cl_2_ under visible light irradiation (Scheme 12).^41^ The use of eosin Y, Mes-Acr^+^ClO_4_^−^, and Ir(ppy)_3_ as a photocatalyst instead of Ru(bpy)_3_Cl_2_ gave the desired product in good yields (51–67%). Hantzsch esters as alkyl radical precursors 35 participated smoothly in oxidative alkylation of active alkenes. These radical initiators underwent a SET oxidation by visible light excited Ru(iii) or K_2_S_2_O_8_, resulting in radical B, pyridine C as the byproduct and a low-valent Ru(ii) complex. The radical species B could be detected in radical trapping reactions including TEMPO, BHT or 1,1-diphenylethene. Then, C was added to the C=C bond of *N*-methacryloyl 2-aryl indole 1 to obtain carbon radical D, which moved through sequential intramolecular radical cyclization to produce intermediate E. Finally, E underwent oxidative deprotonation to furnish the final product 37 (Scheme 13). When aryl substituted Hantzsch esters was applied as a radical precursor, the product was obtained in high yield with dr = 4 : 1 ratio. In Sahoo's work, another ruthenium complex, ([Ru(bpy)_3_](PF_6_)_2_), was utilized for the alkylation/cyclization of *N*-acrylated 2-aryl indoles/benzimidazoles under visible light (Scheme 14).^42^ Interestingly, other photocatalysts, such as *fac*-Ir(ppy)_3_, eosin Y, and 4CzIPN also gave the target product in good yields (61–85%). The replacement of (NH_4_)_2_S_2_O_8_ with aerial O_2_, NFSI, DTBP, or K_2_S_2_O_8_ could be resulted in the formation of the product in 28–64% yield. All parameters; catalyst, oxidant and light had pivotal role in the reaction progress. Dihydroquinazolinones 38 served as alkylaing reagents, resulting in alkyl radicals *via* the action of both the exited-state ruthenium(ii) catalyst and the oxidant under light irradiation. Subsequent radical addition and cyclization, followed by aromatization gave indolo/benzimidazolo[2,1-*a*]isoquinolines bearing a carbon quaternary stereocenter 35, 36. This protocol was also amenable to *N*-methacryloyl anilines and benzamide in the reaction with dihydroquinazolinones.

##### 2.1.2.2 Arylation/cyclization

**Scheme 15:**
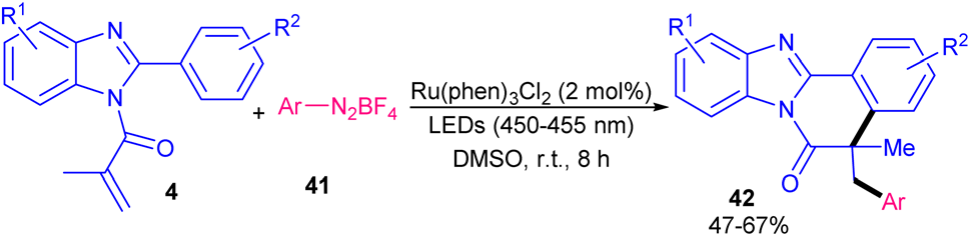
Ru-catalyzed arylation/cyclization of *N*-methacryloyl 2-phenyl benzimidazoles with aryl diazonium salts.

**Scheme 16:**
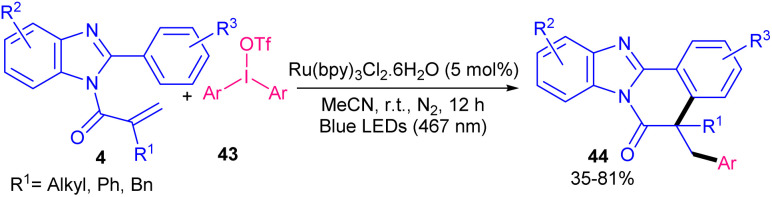
Ru-catalyzed arylation/cyclization of *N*-methacryloyl 2-phenyl benzimidazoles with diaryliodonium triflates.

In 2021, Li and Wang *et al.* developed an arylative cyclization protocol for constructing arylated benzimidazo[2,1-*a*]isoquinolin-6(5*H*)-one scaffolds 42 starting from *N*-methacryloyl 2-aryl benzimidazoles 4 and aryl diazonium salts 41 (Scheme 15).^43^ After evaluating many photocatalysts, including Ru(phen)_3_Cl_2_ (66%), Ru(bpy)_3_Cl_2_ (63%), eosin Y (38%), rose bengal (47%), acridine red (51%), fluorescein (0%) and [Acr^+^-Mes]ClO_4_ (0%), they realized that Ru(phen)_3_Cl_2_ was the most suitable catalyst for this transformation. The plausible mechanism involved photoexcitation of the ground-state Ru(ii) to the excited Ru(ii)*, which was oxidatively quenched by phenyldiazonium salt *via* a SET process to obtain the Ru(iii) and the phenyl radical. Subsequently, the addition of this radical to *N*-methacryloyl 2-phenyl benzimidazole produced another alkyl radical, which underwent intramolecular radical cyclization, SET process and deprotonation toward the assembly of the cyclized product. After a while, another research team reported diaryliodonium triflates as arylating reagents for arylation/cyclization of *N*-acryloyl 2-aryl benzimidazoles under ruthenium catalysis (Scheme 16).^44^ Various diaryliodonium triflates 43 bearing electron-donating and electron-withdrawing groups reacted smoothly with *N*-substituted 2-aryl benzimidazoles 4, leading to the corresponding arylated benzimidazo[2,1-*a*]isoquinolin-6(5*H*)-ones 44 in moderate to high yields (35–81%). Dithiophenyl iodonium triflate gave the desired product in 30% yield. This synthetic method could also be extended to *N*-methacryloyl 2-phenyl indole, providing phenyl-substituted indolo[2,1-*a*]isoquinolin-6(5*H*)-one in 35% yield. It was found that Ru(bpy)_3_Cl_2_ and visible light were inseparable part of this transformation, so that none of the other organophotocatalysts, such as eosin Y, fluorescein, rhodamine B, and rose bengal were successful.

#### 2.1.3 Pd-catalyzed reactions of *N*-methacryloyl 2-aryl indoles/benzimidazoles

##### 2.1.3.1 Alkylation/cyclization

**Scheme 17:**
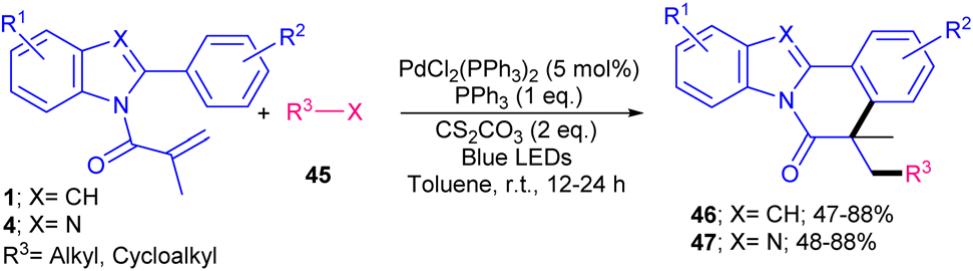
Pd-catalyzed alkylation/cyclization of *N*-methacryloyl 2-phenyl indoles/benzimidazoles.

**Scheme 18:**
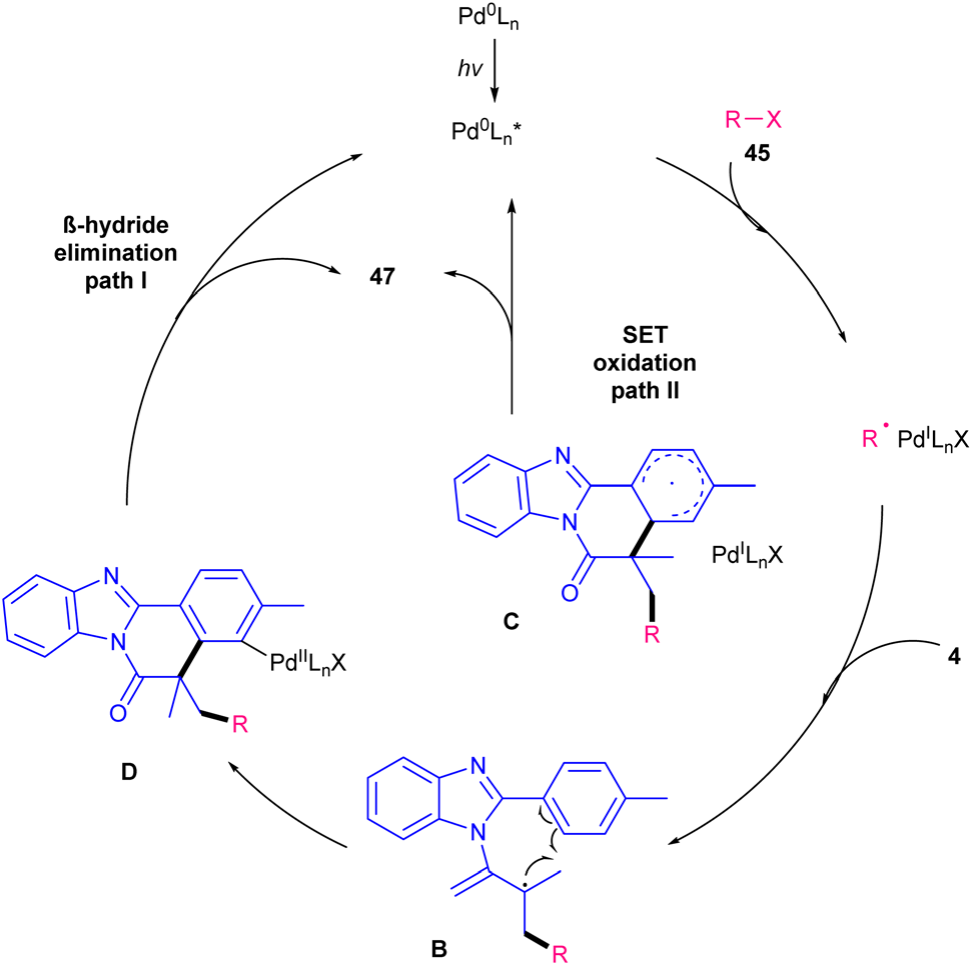
Catalytic cycle for Pd-catalyzed alkylation/cyclization of *N*-methacryloyl 2-phenyl indoles/benzimidazoles.

Visible light-induced palladium-catalyzed alkylation/cyclization of *N*-methacryloyl 2-phenyl indoles/benzimidazoles was reported by Singh and coauthors in 2023 (Scheme 17).^45^ Screening of several palladium catalysts, such as PdCl_2_(PPh_3_)_2_, Pd(PPh_3_)_4_ and Pd(OAc)_2_ showed that PdCl_2_(PPh_3_)_2_ is a superior catalyst, affording alkylated benzimidazo[2,1-*a*]isoquinolin-6(5*H*)-ones in higher yields. Various primary, secondary, and tertiary alkyl halides as alkyl radical initiators 45 reacted smoothly with *N*-methacryloyl 2-phenyl benzimidazoles/indoles 1, 4 affording the corresponding products in good yields. Electron-donating substituents on the aryl ring of benzimidazoles gave the desired products in higher yields compared with electron-withdrawing groups. The lower yield in these cases was attributed to completion of oxidative addition. A Pd(0)/Pd(i)/Pd(ii) catalytic cycle was proposed for this transformation, in which the excitation of the palladium catalyst in the presence of visible light, and subsequent interaction with alkyl halide 45 formed the Pd(i)/alkyl radical hybrid species. These species intercepted the C=C bond of substrate 4 to generate radical B. Then, the aryl ring captured radical B to produce the Pd(i)/alkyl radical C or the Pd(ii) species D. Finally, product 47 was formed either from SET oxidation and aromatization from C (path II), or from β-hydride elimination of D (path I) (Scheme 18).

#### 2.1.4 Fe-catalyzed reactions of *N*-methacryloyl 2-aryl indoles/benzimidazoles

##### 2.1.4.1 Alkylation/cyclization

**Scheme 19:**
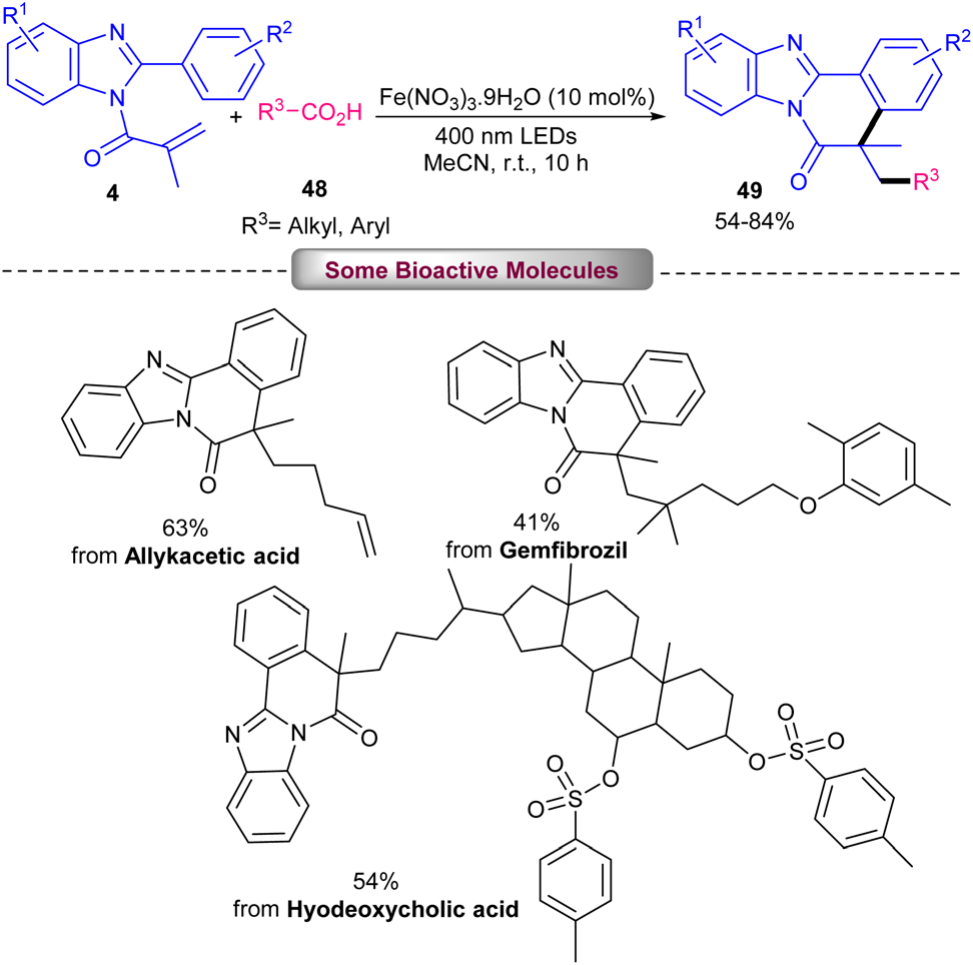
Fe-catalyzed alkylation/cyclization of *N*-methacryloyl 2-phenyl benzimidazoles.

**Scheme 20:**
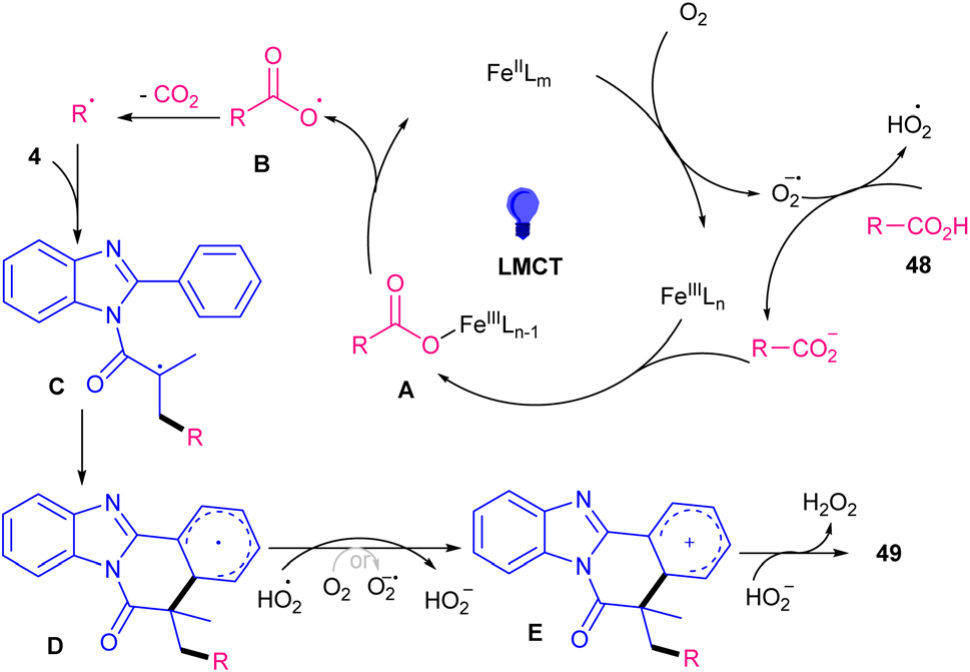
Catalytic cycle for Fe-catalyzed alkylation/cyclization of *N*-methacryloyl 2-phenyl benzimidazoles.

In 2024, Jin and colleagues established alkylation/cyclization of *N*-methacryloyl 2-aryl benzimidazoles 4 using an iron catalyst under light irradiation (Scheme 19).^46^ Just 10 mol% of Fe(NO_3_)_3_·9H_2_O as the sole catalyst in the reaction media could efficiently catalyze carboxylative alkylation of *N*-methacryloyl 2-aryl benzimidazoles in MeCN as a solvent in the presence of 400 nm LEDs. Screening of other iron catalysts; Fe(SO_4_)_3_, Fe(OTf)_3_ and Fe(acac)_3_ resulted in the formation of the products in 25–62% yields. The reaction proceeded through the interaction of Fe(iii) and alkyl carboxylic acid 48 to form complex A. Next, CO_2_ extrusion led to an alkyl radical, which attacked substrate 4 to form radical C. After an intramolecular cyclization, the aryl radical D was obtained, which was oxidized to cation E through a SET step, driven by O_2_ and HO_2_^−^. At last, the product 49 was obtained after the dehydrogenation of HO_2_^−^, together with the formation of H_2_O_2_ as a byproduct (Scheme 20). In order to show versatility of this synthetic method, the tolerance of some natural products containing carboxylic acids were studied.

#### 2.1.5 Ce-catalyzed reactions of *N*-methacryloyl 2-aryl indoles/benzimidazoles

##### 2.1.5.1 Alkylation/cyclization

**Scheme 21:**
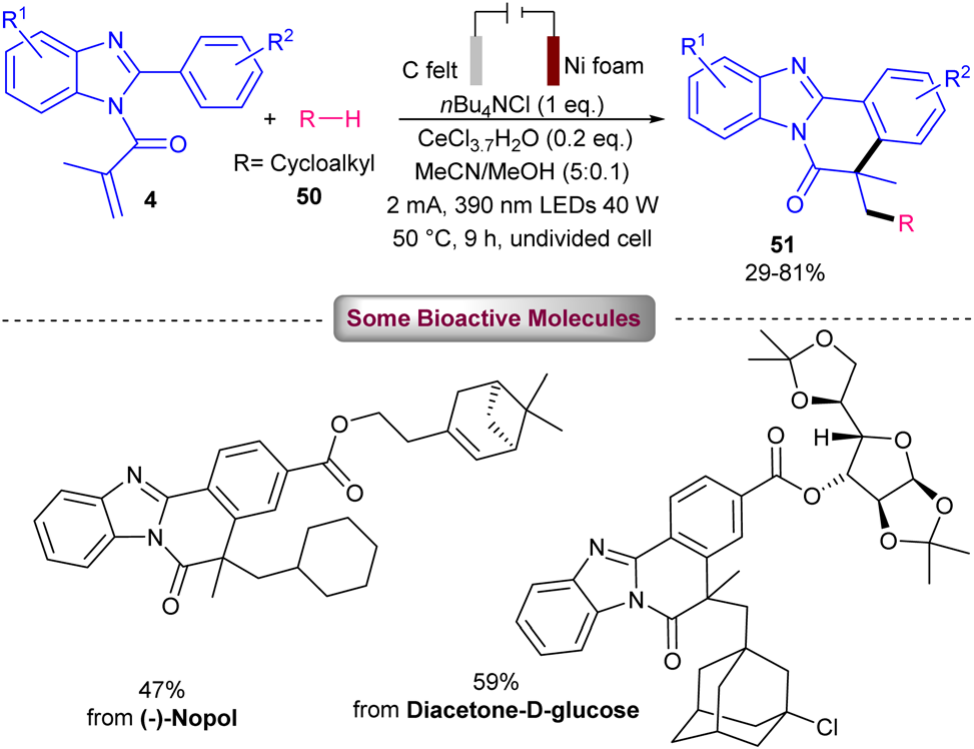
Ce-catalyzed alkylation/cyclization of *N*-methacryloyl 2-phenyl benzimidazoles.

**Scheme 22:**
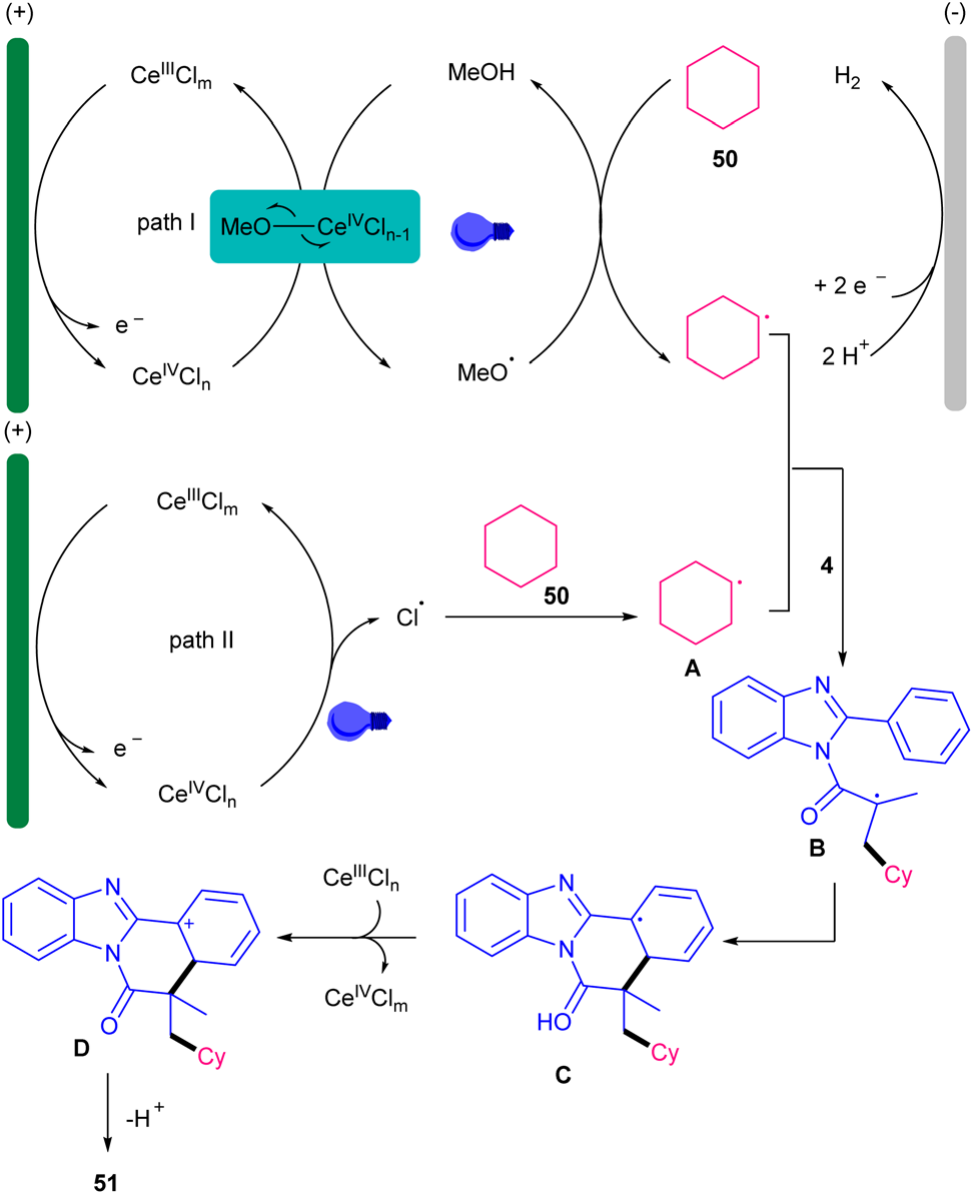
Credible mechanism for Ce-catalyzed alkylation/cyclization of *N*-methacryloyl 2-phenyl benzimidazoles.

**Scheme 23:**
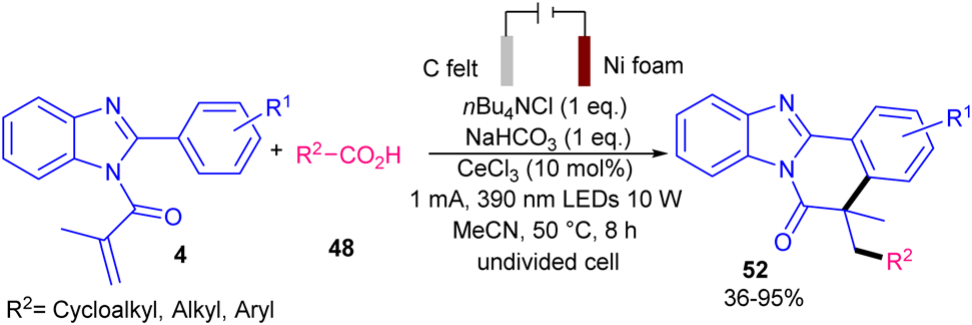
Ce-catalyzed alkylation/cyclization of *N*-methacryloyl 2-phenyl benzimidazoles.

The combination of photochemistry and electrochemistry in the presence of a cerium catalyst can constitute a practical catalytic system for the alkylation/cyclization of *N*-methacryloyl 2-aryl benzimidazoles 4 with unactivated alkanes 50 (Scheme 21).^47^ For this purpose, Zeng and co-workers conducted this transformation in the presence of C felt (+) and Ni foam (−) as electrodes, CeCl_3_·7H_2_O as a catalyst, and 390 nm visible light irradiation in the presence of *n*Bu_4_NCl as an electrolyte in a mixture of MeCN and MeOH as a reaction solvent at 50 °C. However, none of other cerium salts; Ce_2_(SO_4_)_3_, Ce_2_(C_2_O_4_)_3_, Ce_2_(CF_3_SO_3_)_3_ were successful. Various alkylated benzimidazo-fused isoquinolinones were obtained in 29–81% yields. *N*-Methacryloyl 2-aryl indole also represented moderate reactivity, affording the corresponding alkylated indolo-fused isoquinolinones in 43% yield. The preparation of two natural products; (−)-nopol (47%), and diacetone-d-glucose (59%) also was possible using this radical alkylation/cyclization method. This tandem reaction involved the anodic oxidation of Ce(iii) in the presence of *n*Bu_4_NCl and MeOH towards the MeO–Ce(iv)Cl_*n*−1_ complex, which upon ligand-to-metal charge transfer (LMCT) process assisted by visible light gave the methoxy radical. Subsequently, the HAT from 50 to the electrophilic methoxy radical resulted in the cyclohexyl radical A. The radical addition/cyclization of A with 4 furnished intermediate C. Sequential SET oxidation by Ce(iv) and deprotonation delivered product 51 (path I). At the meantime, H_2_ evolution at the cathode consumed the electrons released by the anodic oxidation. The authors attributed that path II involving a chloride radical-initiated HAT process access to A, could also be considered (Scheme 22). The presence of MeOH was found to be vital for the reaction progress and the reaction using other electrolytes; *n*Bu_4_NBr and *n*Bu_4_NI showed low efficiency. After a while, the Zeng group employed these catalytic conditions for decarboxylative alkylation/cyclization of *N*-methacryloyl 2-aryl benzimidazoles 4 using cyclic and acyclic carboxylic acids 48 (Scheme 23).^48^ CeCl_3_ as a catalyst, *n*Bu_4_NCl as an electrolyte, NaHCO_3_ as a base to neutralize the acidic media, MeCN as a solvent and temperature of 50 °C were chosen as a standard condition. The use of both electricity and light were found to be crucial for the process. Primary, secondary and tertiary alkyl carboxylic acids were suitable alkyl radical precursors in radical cascade alkyation/cyclization with radical acceptors 4 bearing electron-poor and electron-rich 2-phenyl portion. This electro-photocatalytic method also represented a high functional group compatibility with respect to carboxylic acids featuring ester, sulfonyl, amide, carbonyl and hydroxyl groups. Again, CeCl_3_ was reported as a catalyst in the alkylation/cyclization reaction of *N*-methacryloyl 2-phenyl benzimidazoles with aliphatic carboxylic acids.^49^ In this reaction, CeCl_3_ (10 mol%), *N*,*N*-diisopropylethylamine (DIPEA) (40 mol%) were used in MeCN at room temperature in the presence two blue-violet LEDs (427 nm).

### 2.2 Photo-mediated metal-free reactions of *N*-methacryloyl 2-aryl indoles/benzimidazoles

Despite many advantages of transition metal catalysis construction of indolo[2,1-*a*]isoquinolin-6(5*H*)-ones and benzimidazo[2,1,*a*]isoquinoline-6(5*H*)-ones, it should be noted that these strategies still suffer from some drawbacks, including the need for expensive metal catalysts, stoichiometric oxidant and high reaction temperatures. In the standpoint of green and sustainable syntheses.^50–53^ The development of direct, convenient and eco-friendly synthetic methods for the construction of structurally diverse indolo/benzimidazo[2,1-*a*]isoquinolin-6(5*H*)-ones is of significance and extremely desirable.

In photoredox-mediated radical cascade functionalization/cyclization reactions, the excited states radicals generated from organocatalysts, are typically highly reactive with short lifetimes because of their inherent instability.

#### 2.2.1 Organocatalyst-catalyzed reactions of *N*-methacryloyl 2-aryl indoles/benzimidazoles

##### 2.2.1.1 Alkylation/cyclization

**Scheme 24:**
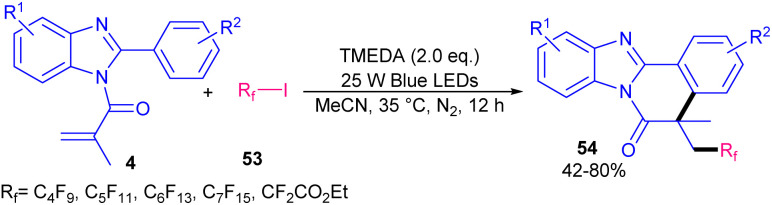
TMEDA-mediated reaction of *N*-methacryloyl 2-phenyl benzimidazoles with perfluoroalkyl iodides.

**Scheme 25:**
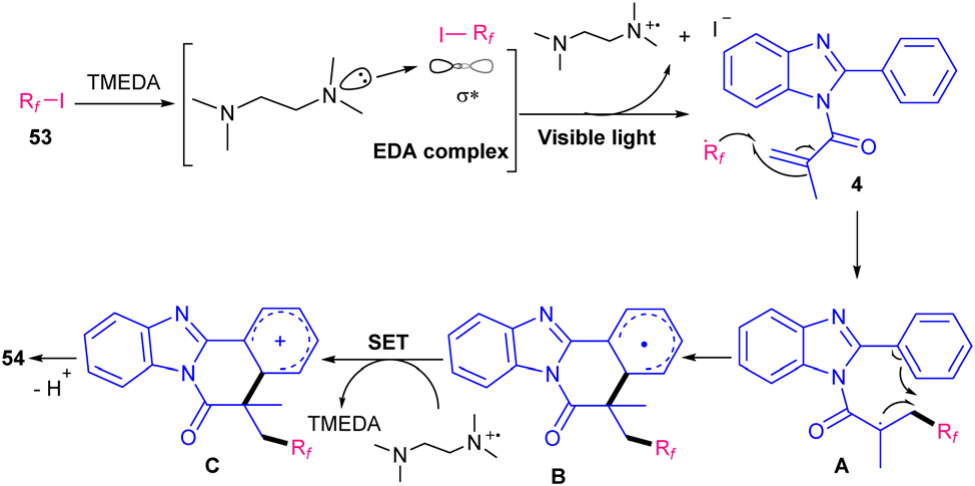
Plausible mechanism for TMEDA-mediated reaction of *N*-methacryloyl 2-phenyl benzimidazoles with perfluoroalkyl iodides.

In 2019, Yu's team employed tetramethylethylenediamine (TMEDA) as a non-metal catalyst to make benzimidazo[2,1-*a*]isoquinoline-6(5*H*)-ones 54 under visible light conditions (Scheme 24).^54^ Various organic bases; TMEDA, 1,4-diazabicyclo[2.2.2]octane (DABCO), 1,8-diazabicyclo(5.4.0)undec-7-ene (DBU), *N*,*N*-diisopropylethylamine (DTPEA), 1,5,7-triazabicyclo[4.4.0]dec-5-ene (TBD), and 1,5-diazabicyclo[4.3.0]non-5-ene (DBN), as well as inorganic bases; Cs_2_CO_3_ and NaHCO_3_ were tested in this reaction. Among them TMEDA was found to be the most suitable base. Perfluoroalkyl iodides as radical initiators with the help of visible light irradiation started this radical addition/cyclization process. EDA complex was generated from TMEDA and perfluoroalkyl iodide 53. 
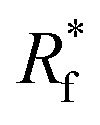
 and TMEDA^+^* were formed under visible light irradiation. Then, 
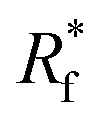
 was added to the C=C bonds of 4 to obtain radical A, which underwent intramolecular cyclization to obtain radical B. The following SET process by TMEDA^+^* furnished carbocation C. Finally, carbocation C was deprotonated to give product 54 (Scheme 25).

**Scheme 26:**
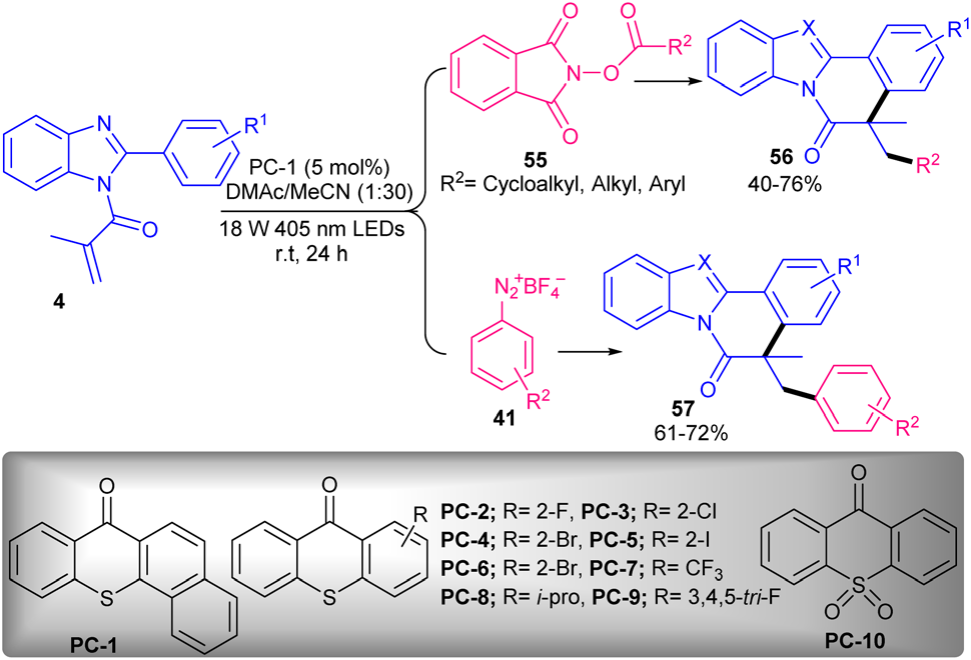
Photocatalysis reaction of *N*-methacryloyl 2-phenyl benzimidazole with *N*-hydroxyphthalimide ester.

**Scheme 27:**
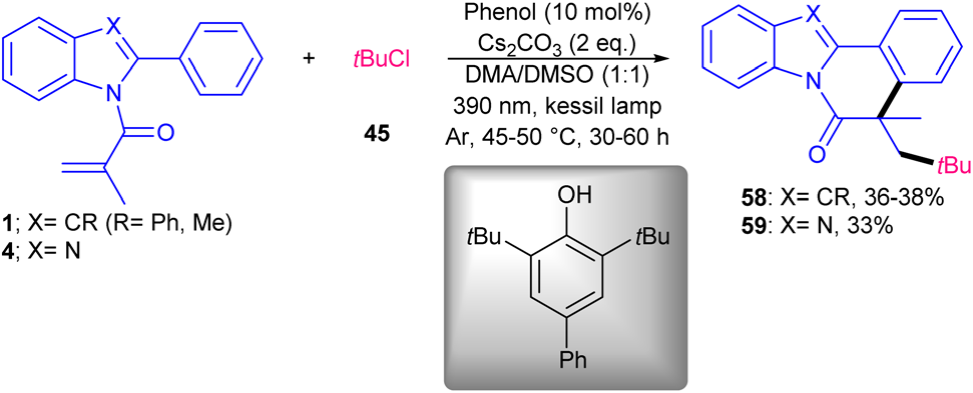
Phenol-promoted alkylation/cyclization of *N*-acryloyl 2-aryl benzimidazoles/indoles.

Various inexpensive and stable aromatic ketones were tested as photocatalysts in radical cascade cyclization reaction of *N*-methacryloyl 2-phenyl benzimidazoles 4 with *N*-hydroxyphthalimide esters (NHPI) 55 or aryldiazonium salts 41 under visible light irradiation (Scheme 26).^55^ Due to the relatively high triplet energy and long triplet lifetime, aromatic ketones found to be effective energy transfer sensitizers. *N*-Hydroxyphthalimide esters were chosen as aryl/alkyl radical precursors. As a redox-active precursor, *N*-hydroxyphthalimide ester can readily accept an electron from reductive species to generate a radical. As aryl precursors, phenyl diazonium salts also showed good compatibility under these conditions. Evaluating of aromatic ketone photocatalysts showed that PC1 has better catalytic activity, affording 69% yield of product, while other photocatalysts PC2–PC10 led to 40–57% yields. The study of the scope of *N*-methacryloyl 2-phenyl benzimidazoles showed that the *ortho*- or *para*-substituted group on the 2-phenyl motif selectively produced a single regioisomer, whereas substrates with a *meta*-substituted group gave a mixture of regio-isomers. For NHPI or aryldiazonium salts, the electronic and steric effects had a minor role in the reaction process. Finally, the utility of this method was demonstrated by the gram-scale synthesis of the product (0.96 gr, 70%). Evaluating of several phenols showed that phenol could be an efficient catalyst for the alkylation/cyclization of *N*-acylacryl amides 4 (Scheme 27).^56^*tert*-Butyl chloride 45 was used as an alkylating reagent and the reaction carried out through *tert*-butyl radical intermediate, which attacked the π-bond of *N*-acryloyl 2-aryl benzimidazoles/indoles. In addition, the alkylation/cyclization of *N*-phenylmethacrylamide derivatives as substrates were well tolerated in this transformation. The presence of phenol catalyst, base and light was essential for this conversion.

**Scheme 28:**
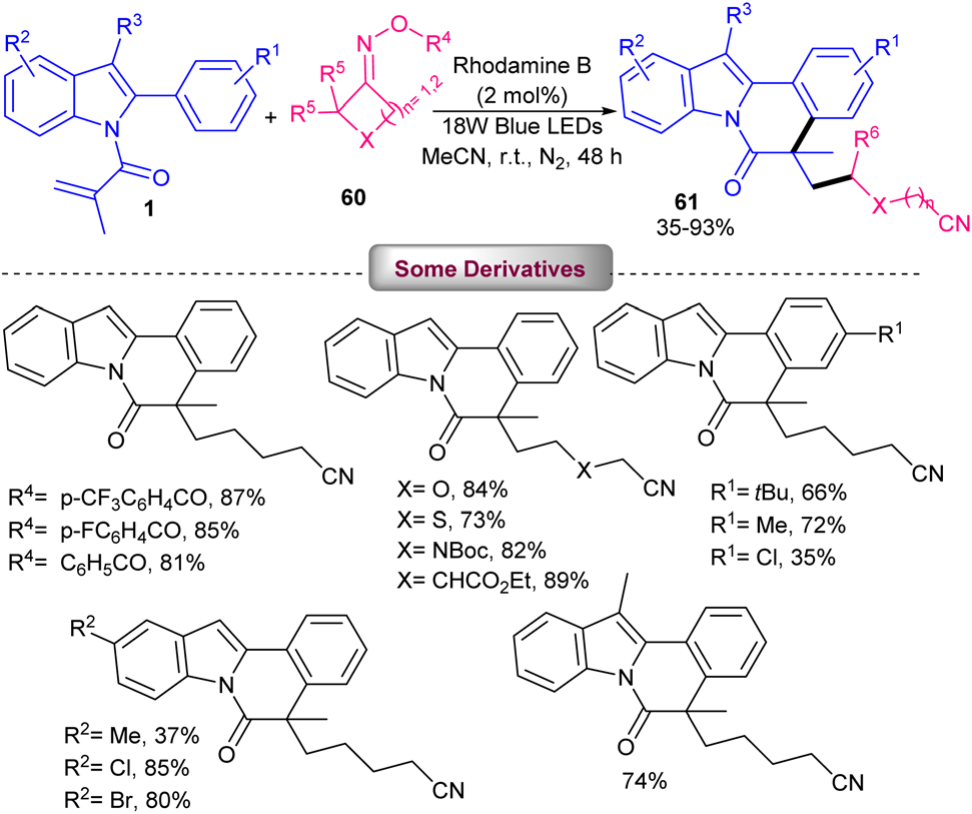
Rhodamine B-catalyzed cyanoalkylation/cyclization of *N*-methacryloyl 2-aryl indoles.

**Scheme 29:**
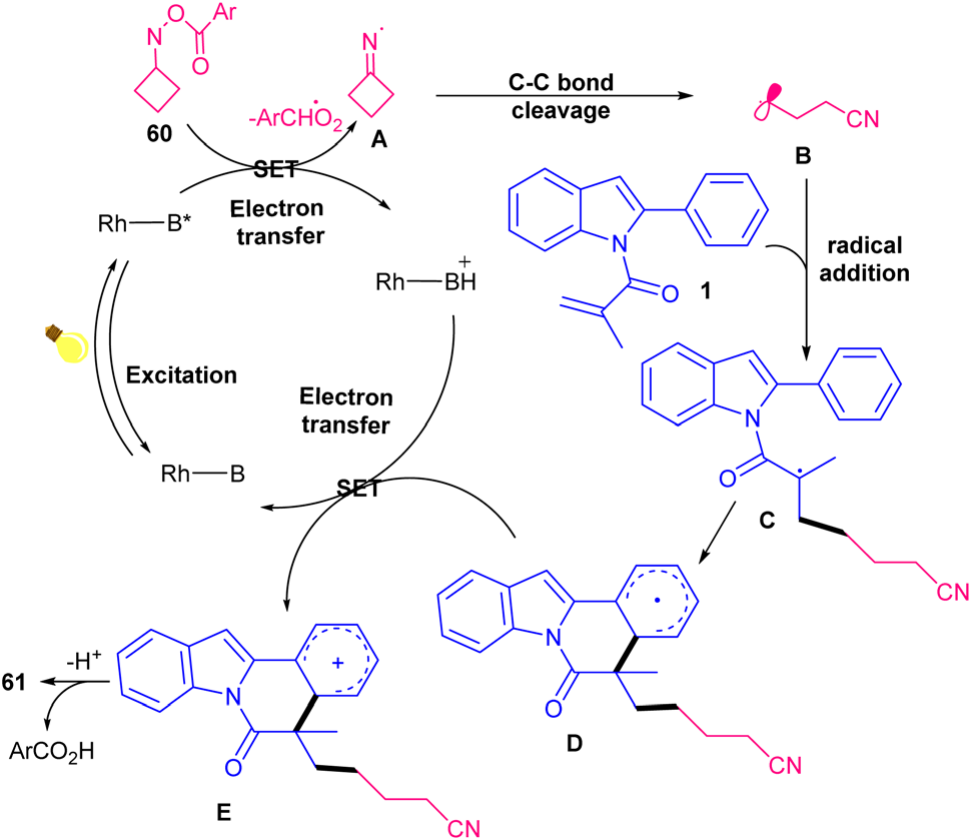
Catalytic cycle for rhodamine B-catalyzed cyanoalkylation/cyclization of *N*-methacryloyl 2-aryl indoles.

**Scheme 30:**
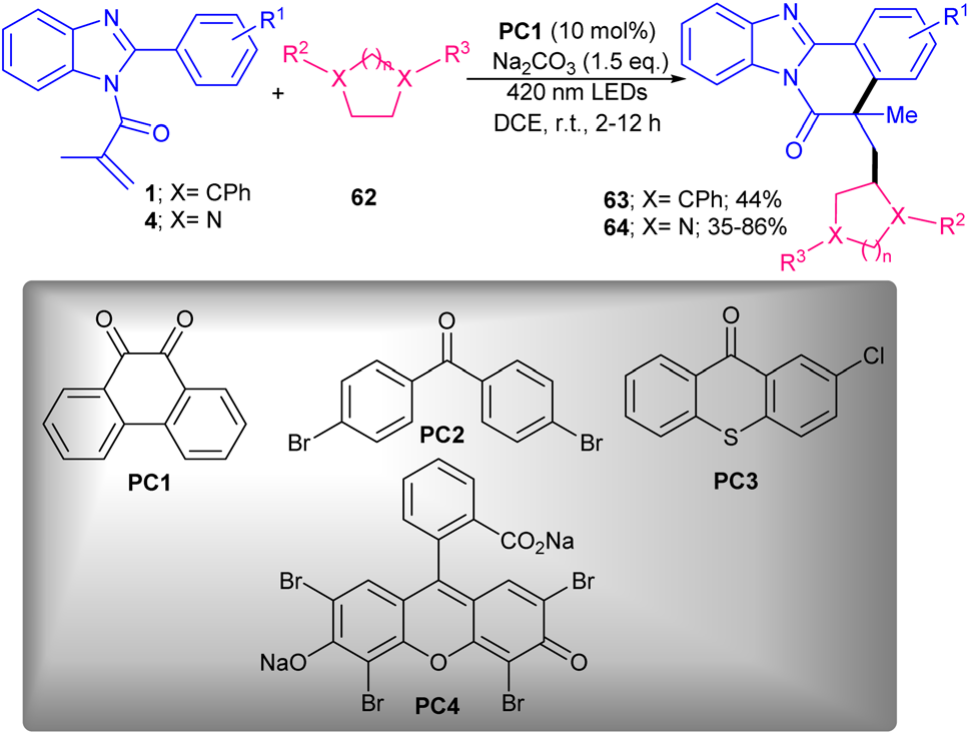
Phenanthrenequinone-catalyzed alkylation/cyclization of *N*-methacryloyl 2-phenyl benzimidazoles.

Rhodamine B was chosen as an appropriate catalyst for photo-mediated cyanoalkylation/cyclization of *N*-methacryloyl 2-aryl indoles 1 with cycloketone oxime esters 60 (Scheme 28).^57^ Eosin B also showed moderate catalytic activity, while rose bengal and methylene blue were not workable. The reaction was found to be proceeded through the formation of the key cyanoalkyl radicals. Firstly, photo promoted conversion of rhodamine B (Rh–B) to Rh–B*, which underwent SET oxidation with cyclobutanone oxime ester 60 to obtain Rh–B^+^, iminyl radical A, and an alkoxy anion. The ring-opening of A produced the highly reactive cyanoalkyl radical B, followed by selectively addition to the C=C bond of *N*-methacryloyl 2-aryl indole 1 to give radical C. Intramolecular radical cyclization of C offered radical D. Then, D was oxidized by Rh–B^+^ through SET to give cation E. In this step, the ground state Rh–B was regenerated for the next cycle. Ultimately, E was deprotonated with the assistance of the alkoxy anion to furnish product 61 (Scheme 29). TEMPO and PhSeSePh both can trap the cyanoalkyl radical B from the reaction media, confirming a radical pathway. In 2024, the alkylation/cyclization of *N*-methacryloyl 2-phenyl benzimidazoles 4 using inactive alkanes and cyclic ethers 62 was established (Scheme 30).^58^ Screening of several organocatalysts; PC1 (61%), PC2 (35%), PC3 (43%), PC4 (0%) in the presence of Na_2_CO_3_ as a base in MeCN as a solvent, showed that organocatalyst PC-1 acted as the best photocatalyst, providing benzoimidazo[2,1-*a*]isoquinolin-6(5*H*)-one derivatives in moderate to high yields (35–86%). Therefore, 10 mol% of phenanthrenequinone (PQ) as an organocatalyst, and 1.5 equiv. of Na_2_CO_3_ as a base were employed in DCE in the presence of 420 nm LEDs at room temperature. Various electron-rich and electron-donor *N*-methacryloyl 2-phenyl benzimidazoles reacted smoothly with cyclic/acyclic alkanes and cyclic ethers under photocatalysis conditions. Phenanthrenequinone acted as a HAT photocatalyst under visible light irradiation and helped in the formation of alkyl radical intermediate. Subsequent radical addition/cyclization delivered the target product.

**Scheme 31:**
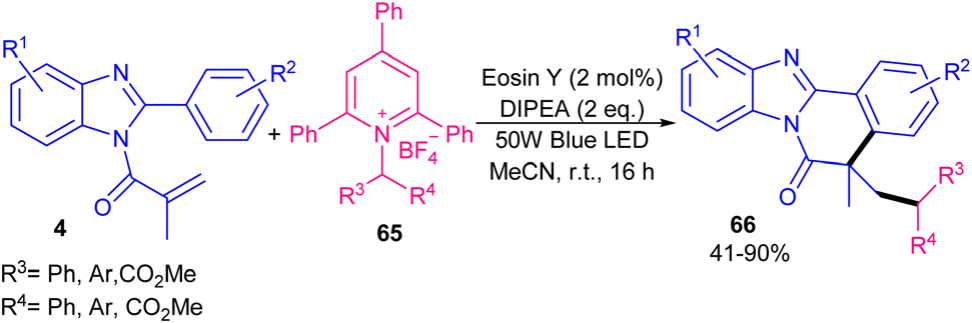
Eosin Y-catalyzed alkylation/cyclization of *N*-methacryloyl 2-phenyl benzimidazoles.

Another organocatalysts-catalyzed alkylation/cyclization of *N*-methacryloyl 2-phenyl benzimidazoles 4 can be carried out in the presence of alkyl amine-derived Katritzky salts 65 as an alkylating reagents (Scheme 31).^59^ Among various organocatalysts; rose bengal, eosin Y, Acr-Mes^+^ClO_4_^−^, and rhodamine B, and metal catalysts; Ru(bpy)_3_PF_6_, Ru(bpy)_3_Cl_2_·6H_2_O and Ir(ppy)_3_, it was found that eosin Y has the best catalytic activity, affording benzo[4,5]imidazo[2,1-*a*]isoquinolin-6(5*H*)-one products 66 in moderate to excellent yields (40–90%). A wide range of electron-rich and electron-poor benzimidazoles reacted well with alkyl amine/amino acid-derived Katritzky salts. The reaction did not influence by steric hindrance and *ortho*-substituted 2-phenyl ring benzimidazoles afforded the corresponding products in good yields. However, *N*-methacryloyl 2-phenyl indoles was not suitable in this protocol, resulting in no product. In general, the mechanism involved single-electron reduction of a redox-active Katritzky salt 2 by excited eosin Y*, resulting in an alkyl radical. Subsequent addition of the alkyl radical to the C=C bond of *N*-methacryloyl 2-phenyl benzoimidazoles 1, followed by an intermolecular cyclization/deprotonation gave the cyclized product 3.

##### 2.2.1.2 Fluoroalkylation/cyclization

**Scheme 32:**
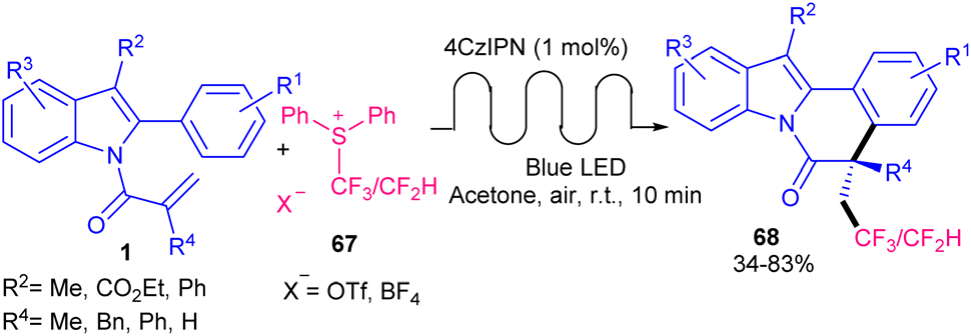
4CzIPN-catalyzed reaction of *N*-methacryloyl 2-phenyl indoles with Ph_2_SCF_3_OTf.

**Scheme 33:**
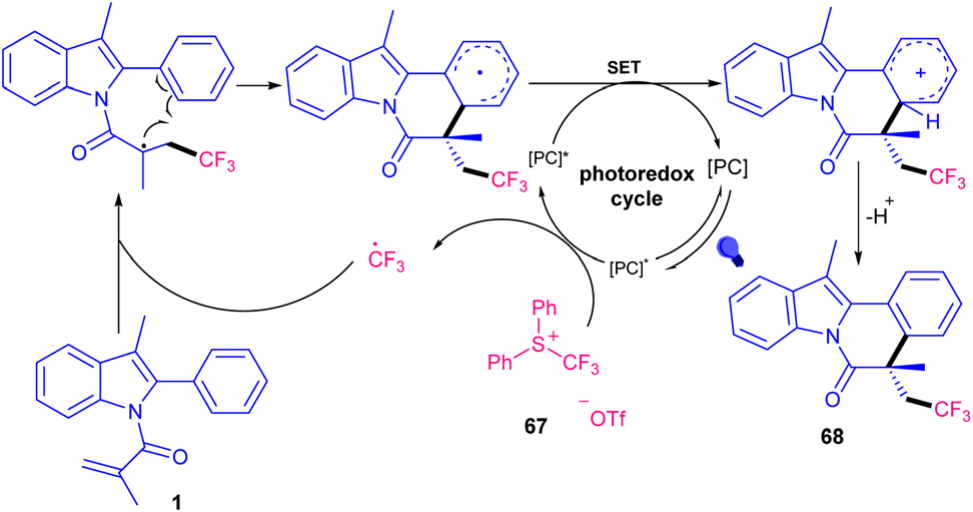
Photocatalytic cycle for 4CzIPN-catalyzed reaction of *N*-methacryloyl 2-phenyl indoles with Ph_2_SCF_3_OTf.

**Scheme 34:**
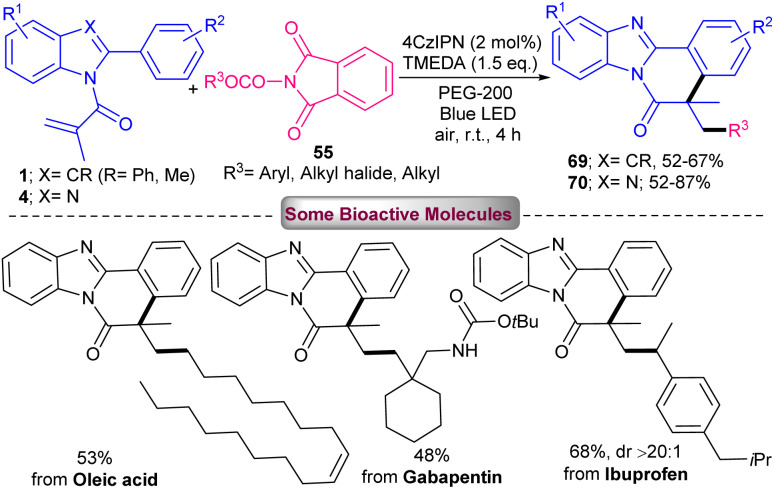
4CzIPN-catalyzed reaction of *N*-methacryloyl 2-phenyl benzimidazoles/indoles with NHPI esters.

A wide range of tri- and di-fluoromethylated indole[2,1-*a*]isoquinoline frameworks 68 were obtained *via* the combination of photochemistry and continuous flow (Scheme 32).^60^ The reaction of *N*-methacryloyl 2-phenyl indoles with Ph_2_SCF_3_OTf occurred in the presence of 1 mol% of 4CzIPN as a photocatalyst in acetone as a solvent in a PFA tube at a flow rate of 100 μL min^−1^ under visible light irradiation at room temperature for 10 minutes. *fac*-Ir(ppy)_3_ also displayed acceptable photocatalytic activity, affording the target product in 51% yield. Mechanistic studies including radical trapping experiment, fluorescence quenching experiments, and Stern–Volmer plots, revealed a radical fluoroalkylation/cyclization sequence. The reaction was initiated by visible light-driven excitation of the photocatalyst PC to PC* together with the reduction of Ph_2_SCF_3_OTf to CF_3_. The generated CF_3_˙ radical then attacked the C=C bond of indole 1 to give a C-radical A, which underwent intramolecular cyclization to form radical B. Subsequently, B interacted with PC^−^ to obtain cation C*via* a SET process. Finally, carbocation C was deprotonated towards product 68 (Scheme 33). The same photocatalyst was employed by another research group in tandem alkylation/cyclization of *N*-methacryloyl 2-aryl benzimidazoles 4 and *N*-methacryloyl 2-aryl indoles 1 with NHPI esters 55 (Scheme 34).^61^ TMEDA as a base involved in the catalytic cycle of 4CzIPN, helping in the formation of alkyl radicals toward the assembly of alkylated benzimidazo[2,1-*a*]isoquinoline-6(5*H*)-ones 70 and alkylated indolo[2,1-*a*]isoquinolin-6(5*H*)-ones 69. Among other organocatalysts; eosin Y, rose bengal, fluorescein, eosin B, and g-C_3_N_4_, only eosin Y provided the desired product in 61% yield, and none of others were effective. The reaction has advantages of the use of NHPI as a reliable and effective alternative for the alkyl radical precursor, polyethylene glycol (PEG-200) as a green solvent, and the performance of the reaction at ambient temperature under air. Furthermore, the synthetic utility of the method was demonstrated by the gram-scale synthesis of the product (0.899 gr, 52%), and the preparation of some bioactive molecules.

**Scheme 35:**
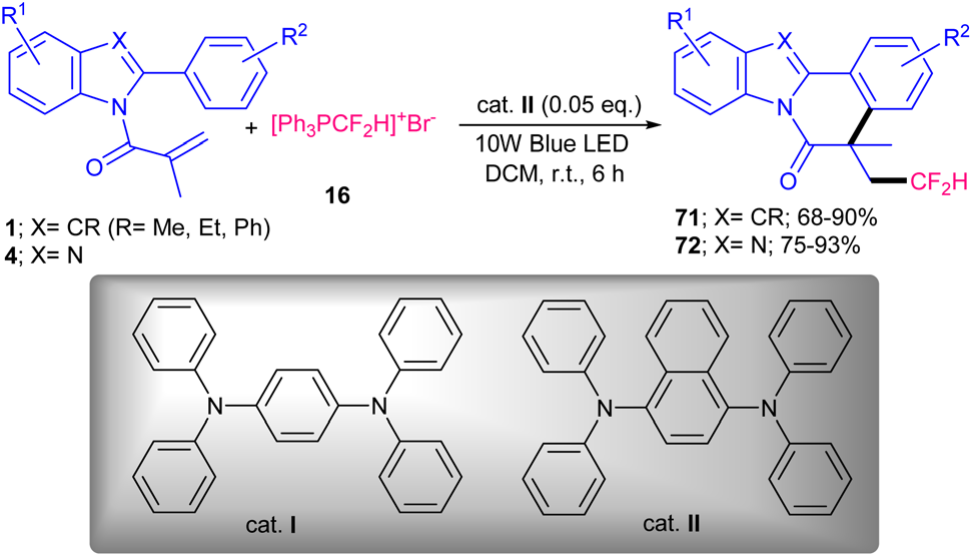
Photocatalysis difluoromethylation/cyclization of *N*-acryloyl 2-aryl benzimidazoles/indoles.

**Scheme 36:**
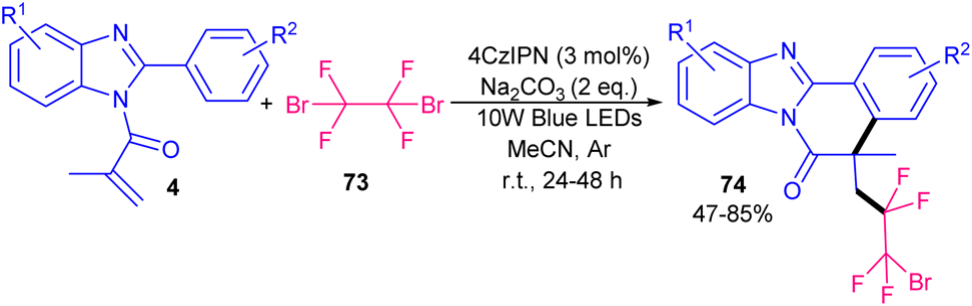
4CzIPN-catalyzed tetrafluoroethylation/cyclization of *N*-methacryloyl 2-phenyl benzimidazoles.

In 2024, Yuan *et al.* developed a photochemical strategy for the difluoromethylation/cyclization of *N*-acryloyl 2-aryl benzimidazoles/indoles 1, 4 (Scheme 35).^62^ In this regard, they investigated various photocatalysts, including 1,2,3,5-tetraki (diphenylamino)-4,6-dicyanobenzene (4DPAIPN), 2,4,5,6-tetrakis(9H-carbazol-9-yl)isophthalonitrile (4CzIPN), eosin Y, Ru(phen)_3_Cl_2_, [RhCp*Cl_2_]_2_, *fac*-[Ir(ppy)_3_], organocatalysts I and II. Among them, 4DPAIPN, *fac*-[Ir(ppy)_3_], I and II led to the desired products in 78%, 90%, 85% and 92%, respectively, while others were not suitable catalysts. Thus, 0.05 equiv. of the catalyst II as the superior catalyst can solely catalyze difluoromethylation/cyclization of *N*-acryloyl 2-aryl benzimidazoles/indoles without the need for any additives. A wide range of CF_2_H-containing benzimidazo[2,1-*a*]isoquinolin-6(5*H*)-ones and indolo[2,1-*a*]isoquinolin-6(5*H*)-ones were well synthesized in good to excellent yields. Catalyst II with the assistance of visible light can catalyze formation of CF_2_H radical that was added to the alkene unit of substrate 1, followed by a cyclization/aromatization step. This protocol featured the gram-scale synthesis of the product (1.098 gr, 88%) and the performance of the reaction under sunlight irradiation that afforded 78% yield. The photocatalyst 4CzIPN represented good catalytic activity for tetrafluoroethylation/cyclization of *N*-methacryloyl 2-phenyl benzimidazoles 4 with 1,2-dibromotetrafluoroethane 73 (Scheme 36).^63^ Just 3 mol% of 4CzIPN provided the product in 46% yield. While *fac*-Ir(ppy)_3_ gave low yield (24%), and [Ru(bby)_3_]Cl_2_ and 3DPA_2_FBN resulted in a trace amount of the product. The elimination of the organocatalyst or light showed the necessity of these parameters in the reaction. The large-scale reaction (3.35 gr, 71% yield) also showed the utility of this synthetic method.

##### 2.2.1.3 Acylation/cyclization

**Scheme 37:**
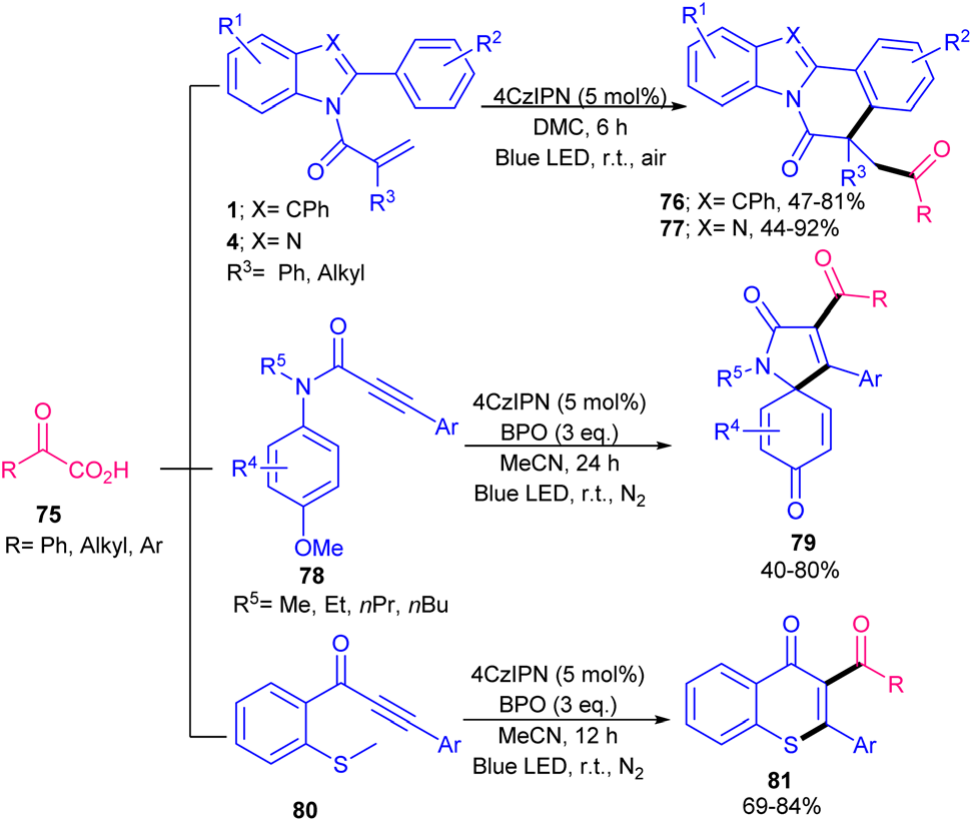
4CzIPN-catalyzed acylation/cyclization of *N*-acryloyl 2-aryl benzimidazoles/indoles.

**Scheme 38:**
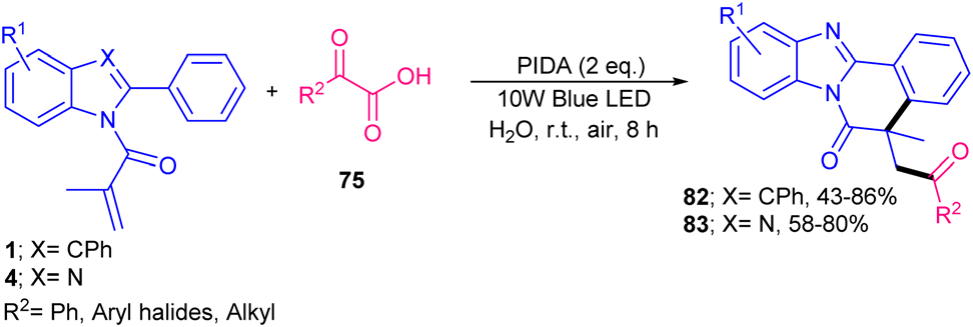
PIDA-promoted acylation/cyclization of *N*-acryloyl 2-aryl benzimidazoles/indoles.

**Scheme 39:**
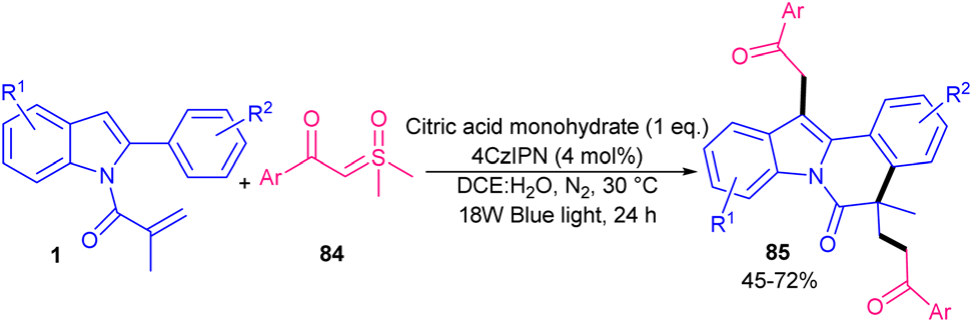
4CzIPN-catalyzed acylation/cyclization of *N*-acryloyl 2-aryl indoles.

In 2021, Chen's research team developed acylation/cyclization of *N*-methacryloyl 2-aryl benzimidazoles/indoles 1 and 4 using α-keto acids 75 as acylating reagents (Scheme 37).^64^ For this purpose, they used 5 mol% of 4CzIPN as an organophotocatalyst and 3 equiv. of BPO as an oxidant in MeCN as a solvent in the presence of visible light to make acylated benzimidazo/indolo[2,1-*a*]isoquinolin-6(5*H*)-ones 76 and 77 under mild conditions. None of other tested organocatalysts; Na_2_Eosin Y, eosin Y, eosin B, and rose bengal were suitable in this reaction. Besides, acylation/cyclization of *N*-arylpropiolamides 78 and methylthiolated alkynones 80 with α-keto acids 75 could produce aroylazaspiro[4.5]trienones 79 and acylated thioflavones 81, respectively, under these organocatalytic system. Radical mechanism was confirmed by using TEMPO and BHT. In addition, Stern–Volmer experiments and cyclic voltammetry indicated that the excited 4CzIPN can oxidize α-keto acids 75*via* SET rather than substrate 1 or 4. A year after, phenyliodine(iii) diacetate (PIDA) was used as a promoter for acylation/cyclization of *N*-acryloyl 2-aryl benzimidazoles/indoles under visible light irradiation (Scheme 38).^65^ A variety of 2-aryl benzimidazoles 4 as well as 2,3-diarylindoles 1 reacted smoothly with 2-oxo-2-phenylacetic acids 75 as acylating precursors, furnishing acylated benzimidazo/indolo[2,1-*a*]isoquinolin-6(5*H*)-ones 82, 83 in moderate to high yields. However, 2-aryl benzimidazoles with R^3^ = H, 2,3-diarylindoles with R^3^ = Me and thiopheneglyoxylic acid were not suitable substrates. The avoidance of transition metals/photocatalysts, performance of the reaction at room temperature, and the use of the green solvent H_2_O represented the advantages of this protocol. α-Keto acids with both electron-donating and electron-withdrawing substituents all reacted well with *N*-acryloyl 2-aryl benzimidazoles/indoles to deliver a new series of 1,4-dicarbonyl-containing benzimidazo/indolo[2,1-*a*]isoquinoline-6(5*H*)-one compounds. The reaction proceeded through a benzoyl radical generated from the I–O bond hemolysis under visible light, followed by CO_2_ extrusion. Subsequent radical addition/cyclization and aromatization yielded the target products. Dual acylmethylation/cyclization of *N*-acryloyl 2-aryl indoles 1 using 3 equiv. of sulfoxonium ylides 84 was reported in 2025 (Scheme 39).^66^ The double molecules of sulfoxonium ylides were subjected to tandem cyclization and C–H coupling reactions with *N*-acryloyl 2-aryl indoles. The radical reaction, which was confirmed by TEMPO and BHT, proceeded in the presence of 4CzIPN as an organocatalyst, citric acid monohydrate as an additive in a mixture of DCE and H_2_O as a reaction solvent under irradiation of blue LED. It is noteworthy that rose bengal, eosin Y and rhodamine B were not suitable organocatalysts, and HCO_2_H, TFA, and TfOH as an acid additive yielded moderate yields.

##### 2.2.1.4 Sulfonylation/cyclization

**Scheme 40:**
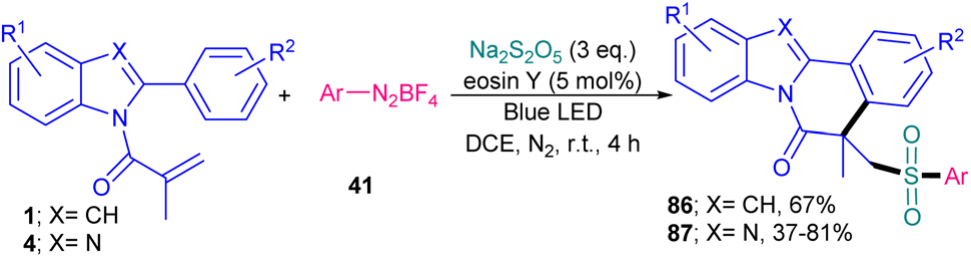
Eosin Y-catalyzed sulfonylation/cyclization of *N*-acryloyl 2-aryl indoles/benzimidazoles.

**Scheme 41:**
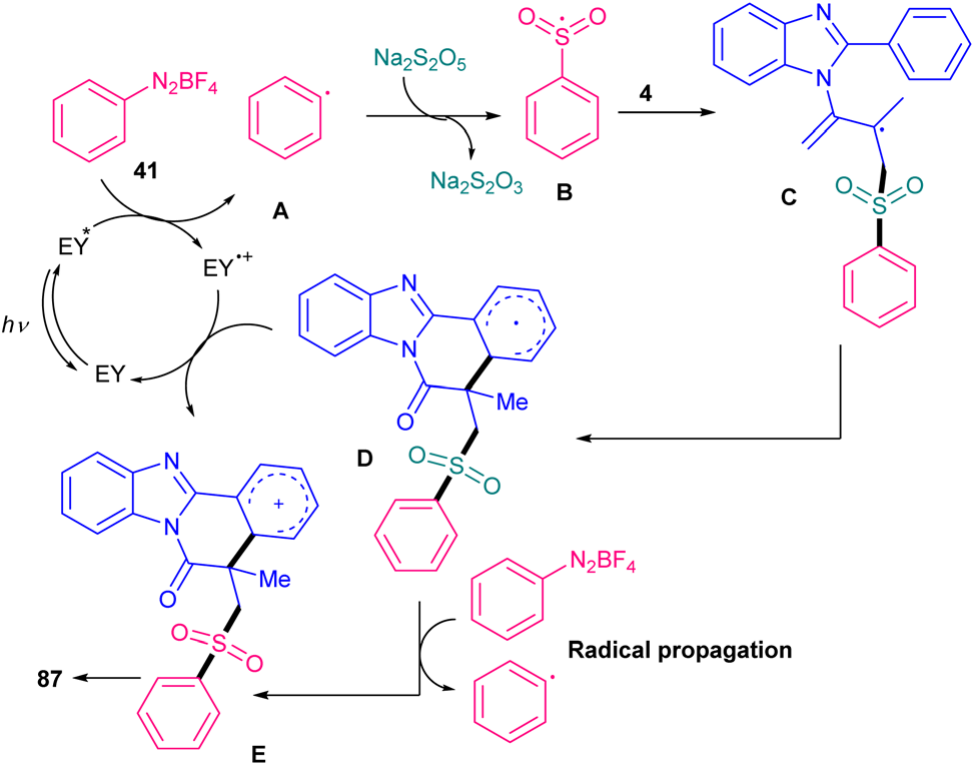
Plausible mechanism for eosin Y-catalyzed sulfonylation/cyclization of *N*-acryloyl 2-aryl indoles/benzimidazoles.

**Scheme 42:**
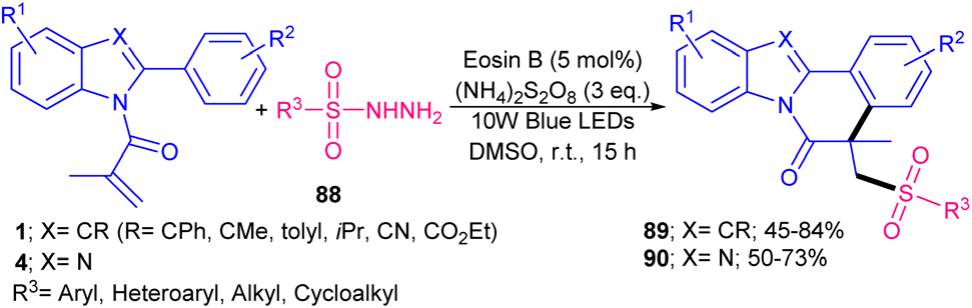
Eosin B-catalyzed sulfonylation/cyclization of *N*-acryloyl 2-aryl indoles/benzimidazoles.

**Scheme 43:**
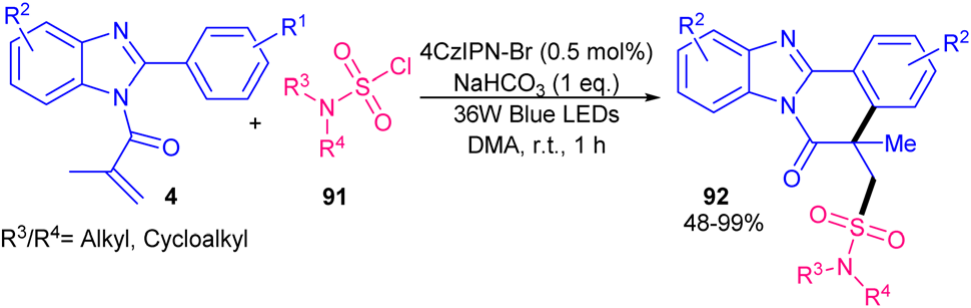
4CzIPN-Br-catalyzed sulfamoylation/cyclization of *N*-acryloyl 2-aryl benzimidazoles.

In 2022, Gupta and co-workers successfully synthesized benzimidazo/indolo[2,1-*a*]iso-quinolin-6(5*H*)-ones through three-component tandem reaction between *N*-acryloyl 2-aryl indoles/benzimidazoles 1, 4 with ArN_2_BF_4_ as an aryl reagent and Na_2_S_2_O_5_ as a SO_2_ surrogate (Scheme 40).^67^ As an organophotocatalyst, eosin Y showed higher catalytic activity (81%) than rose bengal (54%), and 4CzIPN (trace). The sulfonylation/cyclization reaction started with the generation of a phenyl radical from diazonium 41 by the oxidative quenching of the photo-excited catalyst, which was trapped by SO_2_ to give the phenylsufonyl radical B. Further reaction of B with substrate 4 offered the alkyl radical C, which was subjected to the intramolecular cyclization to generate intermediate D. Finally, D was oxidized to E, followed by deprotonation toward the assembly of the product 87. Alternatively, E could be generated by the single electron oxidation in the presence of diazonium salt 41*via* chain propagation (Scheme 41). After a while, Tang *et al.* reported sulfonylation/cyclization of *N*-acryloyl 2-aryl indoles/benzimidazoles 1 and 4 with sulfonyl hydrazides 88 under visible light irradiation (Scheme 42).^57^ For this purpose, they investigated various photocatalysts, such as rose bengal, Na_2_-eosin Y, eosin B, Ru(bpy)_3_Cl_2_·6H_2_O and fluorescein, which produced the desired sulfonylated indolo[2,1-*a*]isoquinolines in good yields (65–75%). Among them eosin B resulted in the best yield (75%). Mechanism was based on sulfonyl radical intermediates, which were generated from photo-induced excitation of eosin B, followed by the reduction of persulfate to the sulfate radical anion. This radical anion could also be directly obtained from (NH_4_)_2_S_2_O_8_ by LED irradiation. Afterward, through the action of sulfate radical anion, sulfonyl radicals were generated from sulfonyl hydrazides, followed by the radical addition/aromatization sequence. 2-Aryl benzimidazoles were also compatible in this reaction, affording benzimidazo[2,1-*a*]isoquinolin-6(5*H*)-ones in 50–73% yields. In 2025, another organocatalyst was applied for sulfamoylation/cyclization of *N*-methacryloyl 2-aryl benzimidazoles 4 with sulfamoyl chlorides 91 as sulfamoylating reagents (Scheme 43).^68^ 4CzlPN (0.5 mol%) and NaHCO_3_ (1 equiv.) effectively catalyzed this reaction under visible light irradiation, producing a new library of sulfamoylated benzo[4,5]imidazo[2,1-*a*]isoquinolin-6(5*H*)-one derivatives in moderate to excellent yields. The use of *fac*-Ir(ppy)_3_ as the catalyst also resulted in moderate product yield (55%), while eosin Y was not effective. It should be pointed out that only aliphatic sulfamoyl chlorides were successful in this reaction system, not those with aromatic motifs. Radical inhibition experiments using TEMPO and 1,1-diphenylethene indicated a radical route and the gram-scale reaction afforded the product in 1.60 gr, with 60% yield.

##### 2.2.1.5 Silylation/cyclization

**Scheme 44:**
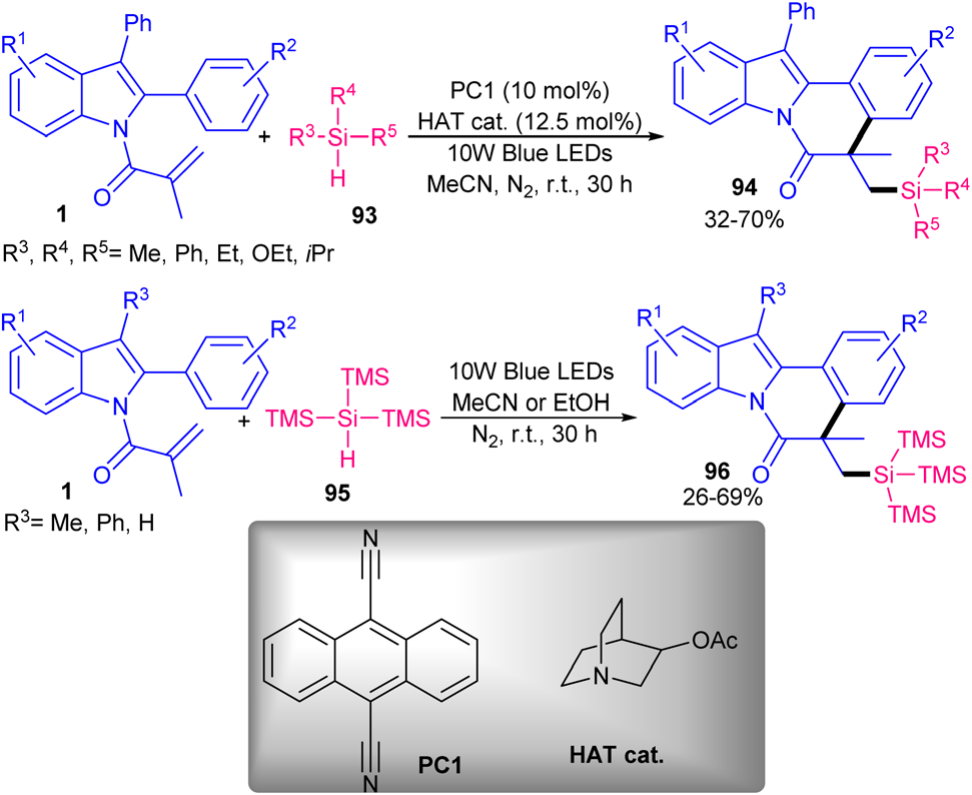
Photocatalysis reaction of *N*-methacryloyl 2-aryl indoles with hydrosilanes.

**Scheme 45:**
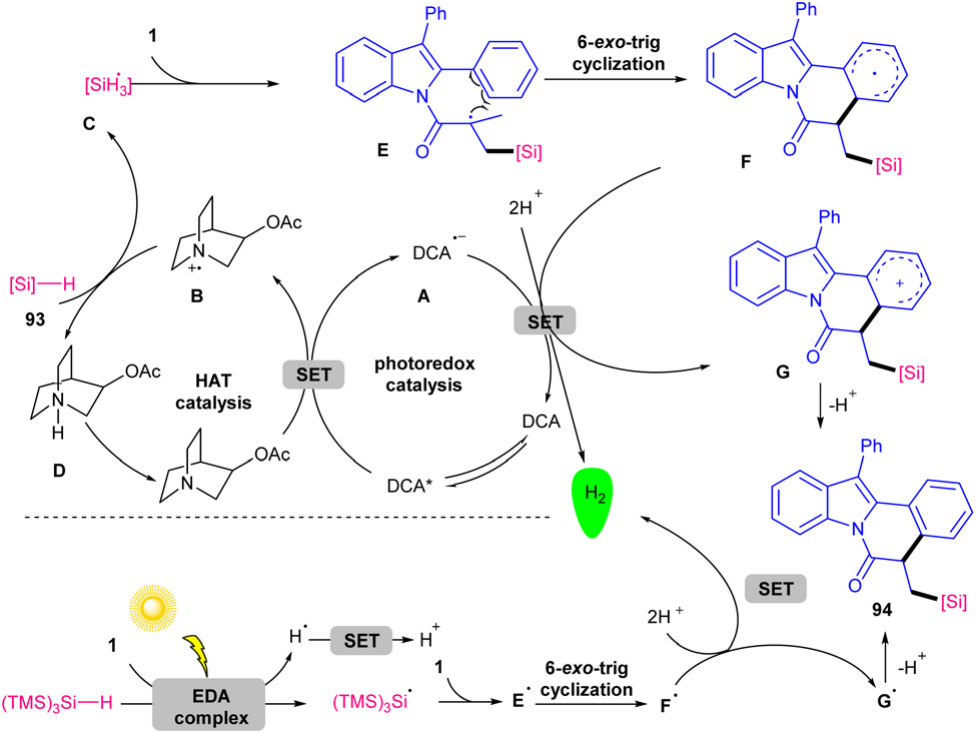
Credible mechanism for photocatalysis reaction of *N*-methacryloyl 2-aryl indoles with hydrosilanes.

The use of two different catalysts; 9,10-dicyanoanthracene (DCA) and 3-acetoxyquinuclidine as organophotocatalyst and hydrogen atom transfer (HAT), respectively, can constitute an elegant system for cyclization between *N*-acryloyl 2-aryl indoles and tri-alkyl/aryl silanes towards silylated indolo[2,1-*a*]isoquinoline-6(5*H*)-ones (Scheme 44).^69^ For (TMS)_3_SiH as a reagent, no catalyst was needed and the transformation carried out only in the presence of 10 W blue LEDs *via* an electron-donor–acceptor (EDA) complex. Both methods have the merits of high atomic economy, metal-free, oxidant-free, H_2_ as by-product, and mild conditions. As depicted in Scheme 45, the mechanism was initiated by the excitation of DCA to DCA*, followed by the reductive quenching by 3-acetoxyquinuclidine to obtain a radical anion A and a radical cation B. Due to its high electrophilicity, B selectively abstracted a H-atom from the more hydridic Si–H bond of hydrosilanes 93 to generate silyl radical C, and cation D. Subsequently, the silylic radical C was added to the C=C bond of indole 1 to form radical E, which was then cyclized *via* 6-*exo*-trig to produce radical F. The SET process between F and A afforded cation G and regenerated DCA. In this process, H_2_ was produced *via* the reduction of two protons. Finally, the deprotonation of G liberated product 94.

#### 2.2.2 Catalyst-free reactions of *N*-methacryloyl 2-aryl indoles/benzimidazoles

##### 2.2.2.1 Acylation/cyclization

**Scheme 46:**
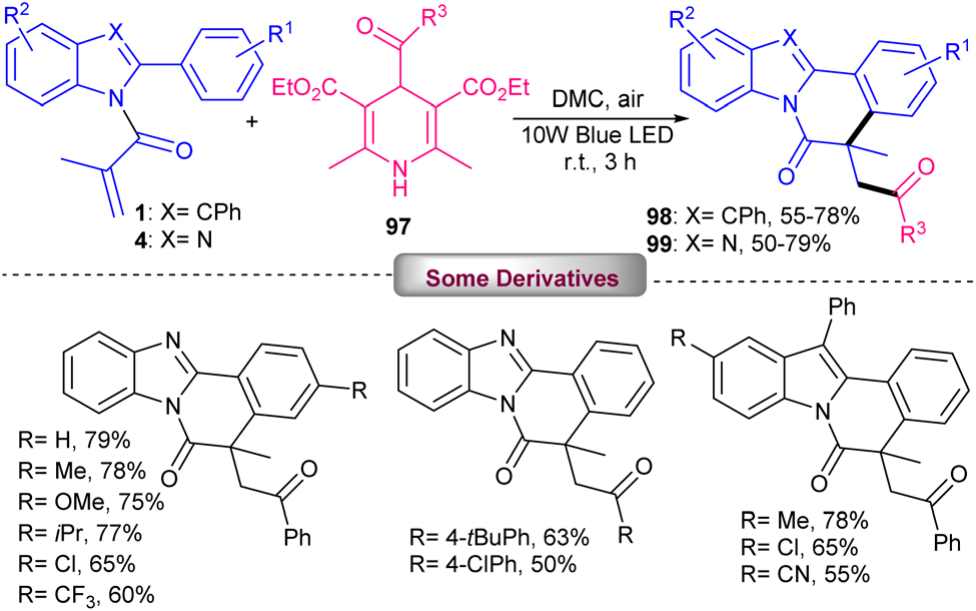
Visible light-mediated acylation/cyclization of *N*-acryloyl 2-aryl benzimidazoles/indoles.

Acylation/cyclization of *N*-methacryloyl 2-aryl benzimidazoles/indoles 4 and 1 with 4-acyl-1,4-dihydropyridines (acyl-DHPs) 97 as acylating reagents can be occurred without any photocatalyst, only by using visible light irradiation at room temperature (Scheme 46).^70^ Among various organic solvents, including MeCN, DMC, DMF, DCE, THF and 2-CH_3_-THF, it was found that the green solvent DMC (dimethyl carbonate) is the best solvent. Several aroylated benzimidazo/indolo[2,1-*a*]isoquinolin-6(5*H*)-ones were achieved in satisfactory yields. Using radical scavengers, TEMPO and BHT, a radical route was proposed for this transformation, involving photo driven formation of benzoyl radicals from acyl-DHPs, followed by the addition to the C=C bond of 2-aryl benzimidazoles/indoles and intramolecular cyclization. This method also could be extended to acylation/cyclization of methylthiolated alkynones, and *N*-methyl-*N*-phenylmethacrylamides with acyl-DHPs.

### 2.3 Electro-mediated reactions of *N*-methacryloyl 2-aryl indoles/benzimidazoles

#### 2.3.1 Alkylation/cyclization

**Scheme 47:**
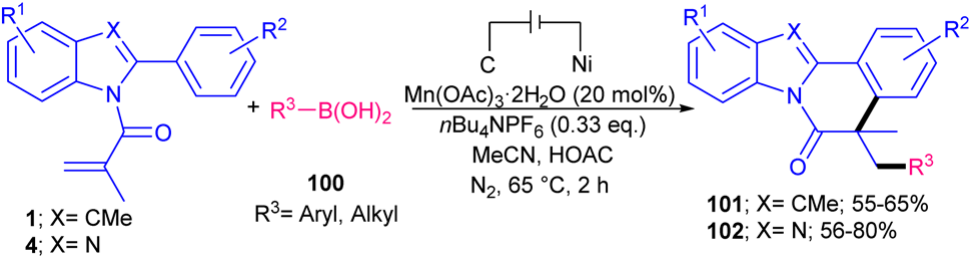
Mn-catalyzed reaction of 2-aryl benzimidazoles/indoles and boronic acids.

**Scheme 48:**
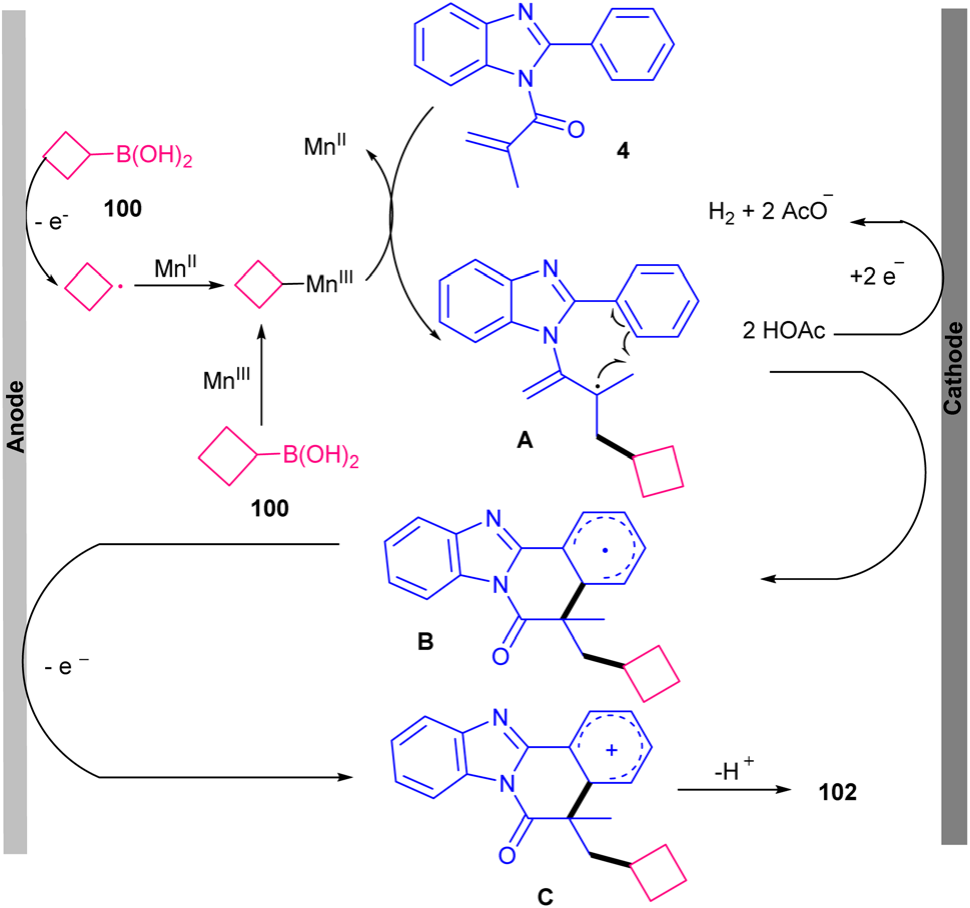
Proposed mechanism for Mn-catalyzed reaction of 2-aryl benzimidazoles/indoles and boronic acids.

Electrochemistry can be used as a green oxidant in Mn-catalyzed cylization reaction of 2-aryl indoles/benzimidazoles 1 and 4 with boronic acids 100 towards indolo/benzimidazo[2,1-*a*]-isoquinolin-6(5*H*)-one scaffolds 101 and 102 (Scheme 47).^71^ Various cyclic and acyclic boronic acids participated in this annulation, leading to moderate to high yields of products. While phenyl boronic acid showed low reactivity and selectivity, affording only 20% yield of product. The presence of Mn(OAc)_3_·2H_2_O was vital, as the reaction without this catalyst resulted in a trace amount of the product even using the Ag(i) catalyst. The manganese catalyst can stabilize alkyl radicals generated from alkylboronic acids. In addition to Mn(OAc)_3_·2H_2_O, Mn(acac)_3_ also gave the desired product in moderate yield (42%). A radical pathway was proposed for this electrochemical cascade cyclization involving the formation of alkyl-Mn(iii) complex from interaction of boronic acid with the Mn(iii), which was converted into the alkyl radical in the anode site. Then, the attack of this radical to the C=C bond of *N*-methacryloyl 2-phenyl benzimidazole 4 produced carbon radical A and the Mn(ii). Further radical cyclization gave intermediate B. The anodic oxidation of B and subsequent deprotonation afforded the product 102. At the cathode, HOAc was reduced to AcO^−^ and H_2_. It was necessary that at first alkylboronic acid 100 to be oxidized at the anode to generate the alkyl radical, and then reacted with Mn(ii) to form the alkyl-Mn(iii) complex (Scheme 48).

#### 2.3.2 Fluoroalkylation/cyclization

**Scheme 49:**
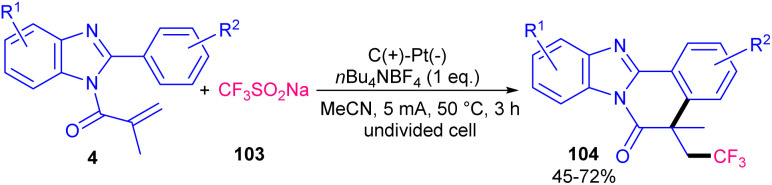
Electrochemical trifluoromethylation of 2-aryl benzimidazoles.

**Scheme 50:**
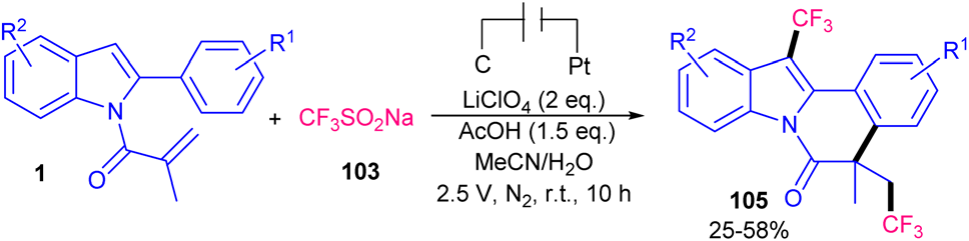
Electrochemical trifluoromethylation of *N*-acryloyl 2-aryl indoles.

**Scheme 51:**
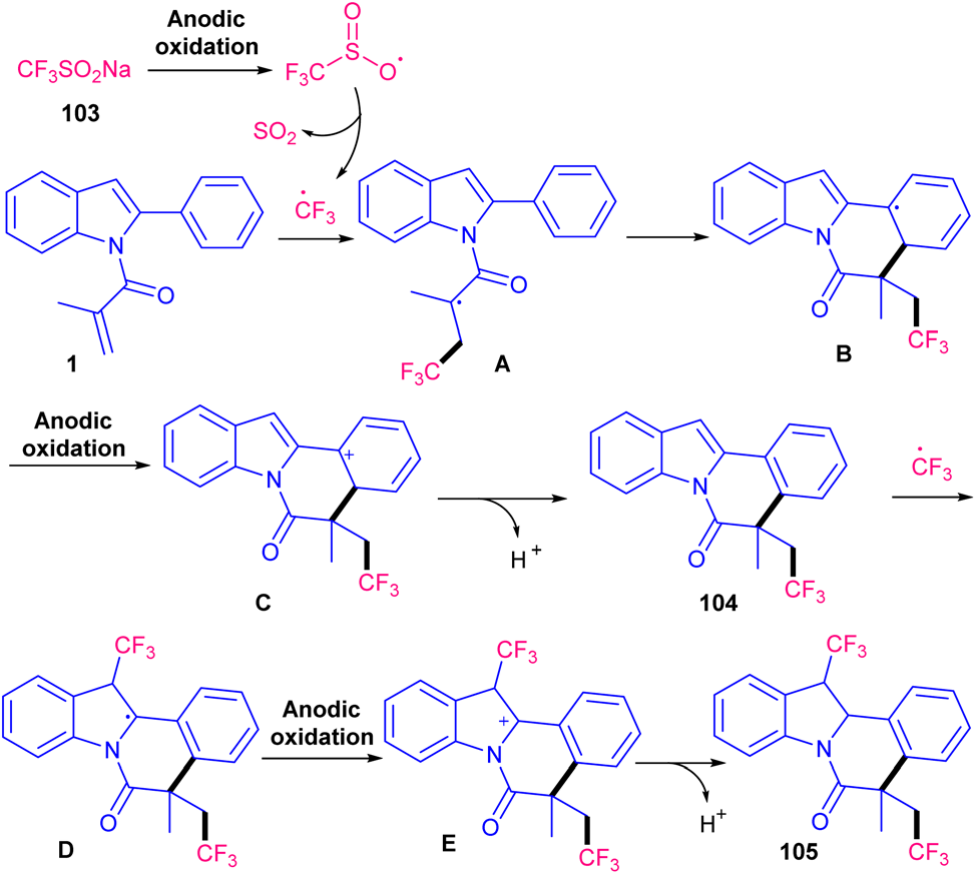
Plausible mechanism for electrochemical trifluoromethylation of *N*-acryloyl 2-aryl indoles.

**Scheme 52:**
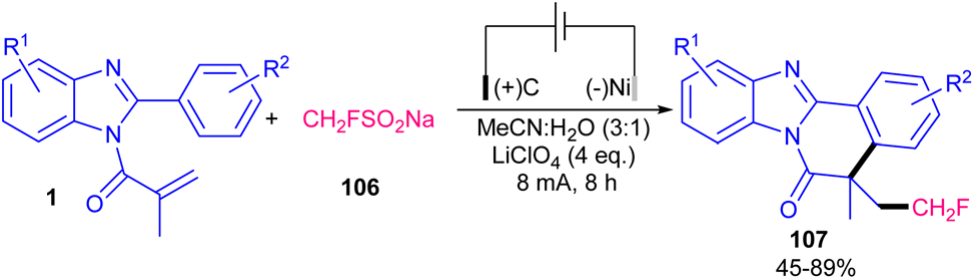
Electrochemical monofluoromethylation of *N*-acryl 2-aryl benzimidazoles.

A metal- and oxidant-free strategy for constructing benzimidazo[2,1-*a*]isoquinolin-6(5*H*)-ones containing CF_3_ moiety from 2-aryl benzimidazoles based on electrochemistey was reported by Zhang *et al.* in 2023 (Scheme 49).^72^ The reaction did not occur at all in the absence of electric current, or replacing CF_3_SO_2_Na with other reagents, such as Togni reagent, and Me_3_SiCF_3_ or even using H_2_O and DMF as the solvent instead of MeCN. *n*Bu_4_NBF_4_ as the electrolyte could enhance the reaction rate more than LiClO_4_, or KF. *N*-Acryloyl 2-aryl benzimidazoles bearing electron-donating groups showed better yields than those with electron-poor substituents. In addition, *para*-substituted benzimidazoles resulted in higher yields than *ortho*- and *meta*-substituted substrates, indicating the steric hindrance as a major factor in influencing the yield. The method was also applicable for the large-scale synthesis of the product (0.792 gr, 62% yield). Cyclic voltammetry and radical trapping experiments suggested a plausible mechanism, in which the CF_3_ radical was generated by anodic oxidation of CF_3_SO_2_Na. This radical reacted with the C=C bond of *N*-methacryloyl 2-phenyl benzimidazole, followed by sequential intramolecular radical cyclization, anodic oxidation and deprotonation. Meantime, hydrogen proton was reduced to H_2_ at the cathode. By using electrochemical method, another research team reported trifluoromethylation/cyclization of 2-aryl indoles 1 with CF_3_SO_2_Na 103 (Scheme 50).^73^ In this protocol, dual trifluoromethylation of 2-aryl indoles occurred, in which mechanistic results and DFT calculations revealed the involvement of sequential formation of CF_3_ radical, the first trifluoromethylation-triggered cyclization and the second trifluoromethylation. Also, intramolecular radical cyclization was found to be the rate-determining step. The reaction was initiated by the anodic oxidation of CF_3_SO_2_Na to form CF_3_SO_2_ radical, which then led to the reactive CF_3_ radical *via* SO_2_ removal. Afterward, CF_3_ radical was captured by 1 to generate radical A. Upon intramolecular radical cyclization of A, another radical intermediate B was generated, which underwent anodic oxidation to produce cation C. The proton abstraction from C delivered compound 104. Subsequently, radical addition between CF_3_ radical and 104 offered new radical D. The next anodic oxidation generated cation E from D, that deprotonated to furnish product 105 (Scheme 51). A transition metal- and oxidant-free electrochemical monofluoromethylation of *N*-acryl 2-aryl benzimidazoles 4 using readily available CFH_2_SO_2_Na 106 was reported in 2025 (Scheme 52).^74^ In this reaction, LiClO4 better acted as an electrolyte compared to *n*Bu_4_NBF_4_ and *n*Bu_4_NPF_6_. Radical trapping reactions using BHT and 1,1-diphenylethylene suggested a radical and SET pathway. Control experiments indicated that the oxidation of the reagent and substrate took place on the anodic site. Thus, similar mechanism to the electrochemical trifluoromethylation of Zhang's work was suggested for this monofluoromethylation of *N*-acryl 2-aryl benzimidazoles towards the synthesis of CFH_2_-functionalized benzimidazo[2,1-*a*]isoquinolin-6(5*H*)-ones.

#### 2.3.3 Sulfonylation/cyclization

**Scheme 53:**
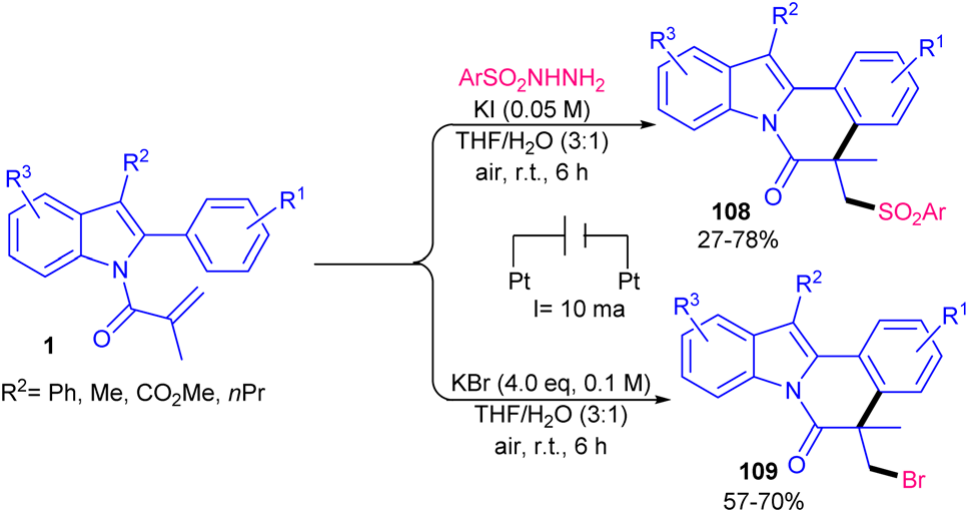
Electrochemical reaction of 2-aryl *N*-acryloyl indoles with sulfonyl chlorides.

The sulfonylation/cyclization of *N*-acryloyl 2-aryl indoles can be carried out under electrolysis conditions (Scheme 53).^75^ A broad substrate scope respect to sulfonyl hydrazides and *N*-acryloyl 2-aryl indoles 1 was investigated in the presence of KI as the only electrolyte in a mixture of H_2_O and THF as a reaction solvent. It is noteworthy that NH_4_I and Bu_4_NBF_4_ were not suitable electrolytes, while TBAI and NaI resulted in inferior yields. Neither H_2_O nor THF were effective as a sole reaction solvent. Constant current was crucial for this transformation. Surprisingly, by changing KI to KBr as an electrolyte and the elimination of sulfonyl hydrazides, the authors isolated different products from the bromination/cyclization of *N*-acryloyl 2-aryl indoles. The study of this reaction in the presence of tosyl iodide also led to the sulfonylated product. NIS and I_2_ showed moderate effectivity in producing product and TEMPO totally inhibited the reaction, indicating a radical mechanism.

## 3 Conclusions

In the recent years, photocatalysis and electrocatalysis have aroused increasing attention of synthetic organic chemists, particularly, in the field of biologically active *N*-heterocyclic compounds. These approaches offer straightforward, and atom-economical routes toward the synthesis of highly functionalized indolo/benzoimidazo isoquinolinone skeletons starting from *N*-acrylated 2-aryl indoles/benzimidazoles. As shown in this review, both metal catalysts and organocatalysts can efficiently catalyze functionalization/cyclization reactions of *N*-acrylated 2-aryl indoles/benzimidazoles *via* a selective manner, although this selectivity was more pronounced in the case of photo-mediated iridium-catalyzed transformation/cyclization reactions. A variety of functionalization reactions, including alkylation, arylation, acylation, carboxylaion, tri/di-fluoromethylation, sulfonylation, and sulfamylation have been reported.

Comparison between the stability of metal complexes with organocatalyst radicals showed that metal complexes can form more stable radicals with higher lifetimes, especially in the case of ruthenium and iridium complexes. This stability allow them to participate in bimolecular electron transfer reactions before deactivation. Also, the lifetime of these exited state species can be extended by appending ligands with long-lived triplet states that can transfer charge to the metal.

The short lifetime of exited states of organic radicals can be a limitation for diffusion-based electron transfer reactions, particularly in photocatalysis, needing high concentrations of reactants. Although some organocatalysts can produce long-lived triplet states when heavy atoms or paramagnetic atoms are involved.

Since photochemistry and electrochemistry are still young and progressing in this field, it is highly desirable to find new free-radical relay reactions for alkenylation, alkynylation, esterification, nitration, halogenation, hydroxylation, cyanation, oxygenation, *etc*. We hope that this review will help researchers to gain inspiration for designing new and green synthetic methods.

## Conflicts of interest

There are no conflicts to declare.

## Data Availability

The authors confirm that the data supporting the findings of this study are available within the references of this review article.
